# Emerging Trends in Silane-Modified Nanomaterial–Polymer Nanocomposites for Energy Harvesting Applications

**DOI:** 10.3390/polym17101416

**Published:** 2025-05-21

**Authors:** Vadakkaveedu Subramanian Niranjana, Sathiyanathan Ponnan, Arvind Mukundan, Arun Anand Prabu, Hsiang-Chen Wang

**Affiliations:** 1 Department of Chemistry, School of Advanced Sciences, Vellore Institute of Technology, Vellore 632014, India; niranjana.vs2022@vitstudent.ac.in; 2 Department of Materials Science, Chulalongkorn University, Bangkok 10330, Thailand; sat.bni@gmail.com; 3 Department of Mechanical Engineering, Advanced Institute of Manufacturing with High Tech Innovations and Research Center for Innovative Research on Aging Society, National Chung Cheng University, Chia Yi County 62102, Taiwan; 4 Technology Development, Hitspectra Intelligent Technology Co., Ltd., Kaohsiung 80661, Taiwan

**Keywords:** silane, coupling agents, nanomaterials, TENG

## Abstract

Nanomaterials (NMs) have gained tremendous attention in various applications in the modern era. The most significant challenge associated with NMs is their strong propensity to aggregate. The chemical surface modification of NMs has garnered notable attention in managing NM dispersion and aggregation. Among the modification approaches, the silane modification of NMs has generated great interest among researchers as a versatile approach to tailoring the surface characteristics of NMs. This review comprehensively examined the recent advancements in silane modification techniques with a focus on triboelectric nanogenerator (TENG) applications. It provides an overview of silane chemistry and its interaction with diverse NMs, elucidating the underlying mechanisms governing the successful surface functionalization process. This review emphasized the silane modification, such as improved mechanical properties of composites, enhanced electrical and thermal conductivity, functional coatings, water treatment, textile industries, catalysis, membrane applications, and biomedical applications, of various NMs. In particular, the role of silane-modified NMs in advancing energy harvesting technologies was highlighted, showcasing their potential to enhance the performance and stability of next-generation devices.

## 1. Introduction

In recent years, advanced nanocomposite (NC) materials have found extensive utility in various commercially significant industries, including automotive, marine coatings, aerospace, and construction. These NCs combine organic materials with inorganic nanomaterials (NMs) using diverse processing methods for superior properties and advanced applications [1]. Materials like metal nanoparticles (NPs), metal oxide (MO) NPs, and carbon-based NMs are commonly employed to craft hybrid NCs [2]. Despite the advantages of NMs, they have few drawbacks. The major challenges are agglomeration, aggregation, and poor dispersity in fluids. NMs possess a high surface area, as well as very strong interaction among the particles that lead to the aggregation or agglomeration of NPs [3]. The agglomeration and aggregation of NMs in hybrid NCs can lead to poor interfacial interaction between the NMs and the dispersing fluids or matrix. The poor dispersion of NM can result in a reduction in the physical and chemical properties of them [4]. To overcome this limitation, several techniques have been adopted, like mechanical mixing, surface functionalization, incorporating hybrid fillers, etc. Among all these methods, the most often used and researched method is surface functionalization [5]. Surface functionalization is described as the covalent or non-covalent binding of organic and inorganic substances onto the surface of NMs, which can facilitate the NM surface to obtain reactive sites for enhancing interfacial bonding to avoid insufficient dispersion and agglomeration in base fluids [6]. Hence, surface-modified NMs have garnered significantly more interest than their unmodified counterparts [7].

NM surfaces have been modified using a variety of techniques. The most frequently and successfully used methods for the functionalization of NMs are silane grafting, polymer grafting, and surfactant-assisted modification methods [8]. Silane is a remarkable coupling agent for the surface functionalization of numerous NMs [9]. Silanes have a surprising bi-functional configuration, which makes them a suitable agent for functionalization. It can act as a bridge to enhance the interfacial interaction between organic and inorganic materials [6]. Many studies have reported the ability of a silane coupling agent in improving the dispersion stability of various NMs in matrices and fluids, thereby enhancing their properties. Wondu et al. (2022) created thermoplastic polyurethane (TPU) composites with strontium titanate (SrTiO_3_) wrapped with carbon nanotube (CNT). The surface of the CNT particles was initially functionalized utilizing 3-aminopropyltriethoxysilane (APTES) for keeping CNT from agglomeration. CNT-covered SrTiO_3_ composites showed a greater dielectric constant and adequate thermal conductivity [10]. Ganguly et al. (2019) [11] conducted a study where they utilized dichlorodimethylsilane to silylate GO, followed by the wet mixing of the modified material as the reinforcement in a polymer matrix. The dispersion of the silane-functionalized graphene oxide (GOF) exhibited a significant advantage over unmodified graphene oxide (GO) in terms of the filler dispersion within the polymer matrix. The inclusion of GOF as a filler resulted in an enhancement of the uniaxial tensile strength compared to GO as a filler [11].

In this review, we explain the surface modification of different NMs, such as fullerene, CNT, graphene, GO, MO NPs, metal, and carbon black (CB), in multiple dimensions using various silanes. Silane has been significantly used in the surface modification of various NMs due to its capacity to serve as a bridging agent between organic and inorganic materials. Surface-modified NMs have the least tendency to agglomerate and, therefore, they can be used in multiple applications. Here, we explored various silanes and their applications in various fields, like textile industries, biomedical applications, catalysis, water treatment, paintings and coatings, energy harvesting, etc.

## 2. Classification of NMs

The term “nanoparticle” refers to particles with diameters of between one and one hundred nanometers that can be in different forms, like tubes, sheets, rods, etc. [12]. NPs display distinct physical and chemical characteristics, like high surface-to-volume ratios, quantum size effect, superior stability, and reactivity, which are all a result of their nanoscale size [13]. Therefore, they have drawn a great deal of interest from scientists and researchers in a variety of scientific fields.

**Figure 1:**
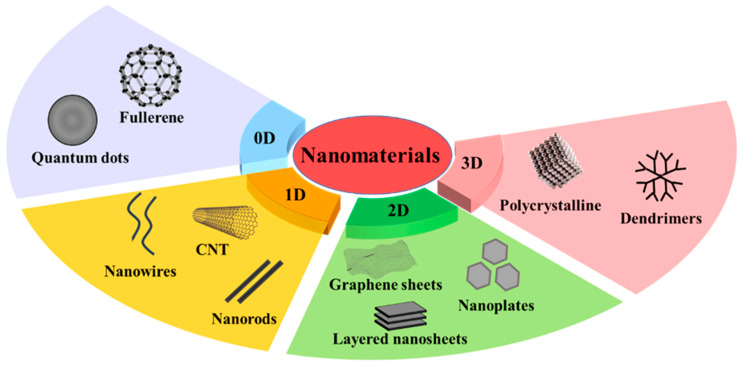
Classification of NMs based on dimensions.

NMs can be divided into zero-dimensional (0D), one-dimensional (1D), two-dimensional (2D), and three-dimensional (3D) categories based on their dimensions. Fullerene, quantum dots, graphene quantum dots, and carbon quantum dots are examples of 0D NMs. Metal, MO, carbon nanotubes (CNT), nanowires, etc., fall under the category of 1D NMs; 2D NMs include graphene, graphene oxide, reduced graphene oxide, etc.; and 3D NMs consist of nanocrystalline material, bulk solids, NCs, etc. [14]. The classification of NMs based on dimensions is shown in Figure 1. Compositionally, NMs can be carbon-based (e.g., graphene, CNTs), organic (e.g., polymeric or lipid NPs), inorganic (e.g., metal or metal oxide NPs), or hybrid systems combining multiple phases, such as polymer-embedded nanocomposites [15,16,17].

## 3. Synthesis of NMs

NMs can be produced through physical, chemical, and biological methodologies, which are further classified into top-down and bottom-up approaches. There exists a diverse range of physical, chemical, and biological techniques that can be utilized for the synthesis of NMs in various dimensional forms, including 0D, 1D, 2D, and 3D structures. These methods enable a fabrication that allows control of their size, shape, structure, and properties.

### 3.1. Physical Methods

#### 3.1.1. Ball Milling

Ball milling is a mechanical method for producing NMs and is classified as a top-down approach. It involves grinding materials using steel balls in a rotating container. This process is valuable for creating NPs and NCs with controlled properties, size, and distribution, making it vital in nanotechnology and materials science research [18]. This approach is the favored choice for synthesizing intermetallic NPs [19]. This method is preferred for blending aluminum with magnesium and carbon in order to alter its chemical properties and combustion behavior [20]. Zou et al. employed a straightforward ball milling strategy to prepare a large-scale batch of V_2_O_5_ NPs with oxygen vacancies in their study [21].

#### 3.1.2. Chemical Vapor Deposition (CVD)

CVD is a bottom-up NM fabrication technique in which vaporized precursor gases react on a substrate’s surface, creating thin films, nanowires, or NPs. CVD enables precise control over material composition, thickness, and structure, making it vital in semiconductor manufacturing, nanoelectronics, and materials research [22]. It helps to generate NPs with precisely controlled surface morphology [23]. The favored technique for producing gas-sensitive SnO_2_ nanorods is through aerosol-assisted chemical vapor deposition [24]. CVD is a highly favored approach for carbonaceous NM synthesis due to its relative simplicity and the substantial degree of control it provides [25]. Over time, this field has grown, and, now, the method can be confidently applied at a larger scale to produce high-quality graphene materials [26].

#### 3.1.3. Laser Ablation

Laser ablation is a NM synthesis technique that employs laser energy to vaporize or ablate a target material, and it is classified as a top-down approach. The resulting vapor condenses into NPs or thin films on a substrate. Laser ablation enables precise control over NM size, composition, and structure, making it valuable in nanotechnology and materials science research [27]. It is a relatively straightforward and efficient method for generating a substantial quantity of small nano-sized particles in suspension form, and Rivera-Chaverra et al. were able to create iron oxide NPs through laser ablation [28].

#### 3.1.4. Arc Discharge

Arc discharge is a top-down method for producing NMs, such as CNT and fullerenes, by vaporizing a target material, often graphite, in an electric arc. The vaporized carbon atoms reassemble into nanoscale structures as they cool and condense [29]. In their study, Zhang et al. explored, focusing on the influence of buffer gas and pressure, the mechanism of carbon NM formation using the DC arc discharge method [30]. Gojny et al. produced multi-walled CNTs (MWCNTs) by arc discharge method, and the resulting composite was embedded in the epoxy resin.

### 3.2. Chemical Methods

#### 3.2.1. Chemical Reduction

Chemical reduction synthesis is a widely used bottom-up method for creating NMs, particularly metallic NPs. It involves a reduction in the metal ions that are in a solution by adding a reducing agent, leading to the formation of NPs. A reducing agent, like sodium borohydride, is employed in an aqueous solution for the synthesis of metal NPs. This approach allows precise control over particle size, shape, and properties, making it valuable in diverse applications [31]. Zhang et al. synthesized copper NPs utilizing potassium borohydride as the reducing agent [32].

#### 3.2.2. Sol–Gel Process

The sol–gel process is a versatile bottom-up technique for synthesizing NMs. It involves converting a precursor sol (a colloidal suspension) into a gel and then into a solid material. This method enables the production of NPs, thin films, and coatings with proper control over composition, structure, and properties, which is valuable in various industries [33]. This method is employed for the fabrication of zinc peroxide (ZnO_2_) nanostructures [34].

#### 3.2.3. Hydrothermal/Solvothermal Synthesis

Hydrothermal and solvothermal synthesis are bottom-up methods for creating NMs under high-temperature and high-pressure conditions in aqueous or organic solvents, respectively. These techniques allow for precise control over NP size, morphology, and crystallinity, making them essential in producing a wide range of NMs for applications in chemistry, materials science, and nanotechnology [35]. Yang et al. prepared high-quality, crystallized, monodispersed nanocrystals in their synthesis of silver (Ag) NPs [36].

#### 3.2.4. Emulsion Techniques

Emulsion techniques are bottom-up methods that involve creating nanoscale droplets of one liquid within another immiscible liquid. These methods are employed to fabricate NMs, such as NPs or encapsulated materials, with a controlled size and structure. Emulsion techniques are widely used in fields like pharmaceuticals, cosmetics, and nanotechnology for tailored material synthesis [37]. Li et al. effectively produced Pt, Sn NPs supported on carbon, for application in direct ethanol fuel cells using an enhanced micro-emulsion method [38].

### 3.3. Biological Methods

#### 3.3.1. Plant-Mediated Synthesis

The biosynthesis of NPs using plant extracts has gained significant attention as an eco-friendly, cost-effective, and sustainable alternative to conventional physical and chemical synthesis methods [39]. It is a green chemistry approach that uses plant extracts, which act as natural reducing and stabilizing agents, to convert metal ions into NPs. The process is simple, involving the mixing of plant extracts with a metal salt solution, where the phytochemicals reduce the metal ions and stabilize the resulting NPs [40,41]. Ansari et al. synthesized Al_2_O_3_ NPs from lemon grass leaf extract, which were spherical in shape, at the size of 9–180 nm [42].

#### 3.3.2. Bacteria in Synthesis of NPs

The bacteria-mediated synthesis of NPs represents an innovative and environmentally friendly approach that harnesses the metabolic capabilities of microorganisms to produce various types of NPs. Certain bacterial species possess the unique ability to reduce metal ions into NPs through either intracellular or extracellular mechanisms. Bacteria have adapted to metal-rich environments and can neutralize metal ions through mechanisms such as reduction or precipitation, leading to the formation of metal nanoclusters. Bacteria offer the advantage of rapid growth cycles and facilitate straightforward extraction of the synthesized NPs [43]. The bacterial strain *Pseudomonas stutzeri* AG259, originally obtained from silver mine environments, has been reported to biosynthesize silver NPs. Similarly, magnetotactic bacteria, such as *Magnetospirillum magneticum*, are known to generate magnetic NPs composed of Fe_3_O_4_ [44].

#### 3.3.3. Fungi Mediated Synthesis

Recent advancements have seen a growing interest in exploring fungi as biological agents for the production of various NPs [45]. Fungi have emerged as preferred biological systems for NP synthesis due to several unique advantages. One key benefit is their ability to secrete large quantities of extracellular enzymes and proteins that can effectively reduce metal ions into NPs while also stabilizing them [46]. Fungal-derived NPs have shown excellent antimicrobial, anticancer, and catalytic properties, with potential applications in medicine, agriculture, and environmental remediation [45]. The fungus *Fusarium oxysporum* is frequently employed in NP biosynthesis. It is capable of producing extracellular gold (Au) NPs, typically ranging from 20 to 40 nm in size, with spherical or triangular morphologies. These NPs remain uniformly dispersed and show no notable aggregation, even after one month of storage [47].

#### 3.3.4. Yeast in the Synthesis of NPs

Recent studies have demonstrated the remarkable potential of yeasts as biofactories for NP synthesis, offering an eco-friendly alternative to conventional chemical methods. Varying culture conditions, such as the pH, temperature, and incubation time, can significantly influence the NP size, morphology, and crystallinity. Extremophilic yeast strains obtained from acid mine drainage environments have demonstrated an impressive ability to synthesize Ag and Au NPs. These metal-resistant yeasts can thrive in the presence of up to 1.5 mM silver ions and 0.09 mM gold ions, generating extracellular NPs sized between 20 and 100 nm [48].

## 4. Surface Modification Techniques of NMs

Surface modification stands as one of the widely embraced approaches for averting the agglomeration of NMs. This method offers the capability to augment the stability of NMs within suspensions and promote their harmonious integration with organic matrices or biological settings. To modify the surfaces of MO NMs, the physicochemical interactions between NMs and modifiers can be harnessed. In the following section, we will deal with the details of both physical and chemical techniques employed for the surface modification of NMs.

### 4.1. Physical Techniques

Physical surface modification is a straightforward method to enhance the stability of NMs by applying ionic or polymeric surfactants to the nano surface. Surfactants have hydrophobic tails and hydrophilic heads, and their hydrophilic groups adsorb onto NM surfaces via electrostatic interactions or chemical bonding. This reduces interparticle interactions, minimizing interfacial forces, lowering surface tension, and decreasing NM aggregation. However, physically modified NMs exhibit thermal and solvolytic instability due to weak van der Waals forces and hydrogen bonds on their surfaces [49]. Typical surfactants employed in the surface modification of NMs encompass sodium dodecyl sulphate (SDS), cetyltrimethylammonium bromide (CTAB), and polyvinylpyrrolidone (PVP) alongside a variety of other polymers, amphiphiles, and ligands [50]. Li et al. discovered that ionic surfactants like SDS can significantly enhance the stability of TiO_2_ NPs while reducing aggregation [51]. CTAB-coated Fe_3_O_4_ NPs were investigated for quantifying trace concentrations of 15 priority polycyclic aromatic hydrocarbons (PAHs). Their study demonstrated that CTAB-Fe_3_O_4_ NCs, serving as solid adsorbents combined with ultra-performance liquid chromatography and fluorescence detection FLD, offered excellent performance in analyzing trace PAHs in water [52]. Lee et al. fabricated a 3D network film composed of stearic acid-coated CNTs and cellulose nanofibers (CNFs). Octylamine, a surfactant, was used to bind CNFs and CNTs. The film with the network, filled with 70 wt.% filler, exhibited a 259% improvement in thermal conductivity compared to pure CNF, surpassing the film with unmodified CNTs as fillers [53]. In another study by Heltina et al., the addition of surfactant (CTAB) to CNTs aimed to enhance CNT solubility in water, thereby introducing functional groups with anionic and cationic characteristics to the nanotube surfaces [54]. Salihi et al. studied the removal of Ni (II) ions from aqueous solutions using GO and SDS-GO. The adsorption capacity of the adsorbent significantly increased (from 20.19 to 55.16 mg/g) due to surface functionalization by SDS [55]. Wu et al. [56] prepared PVP-coated GO and investigated its effects on the desalination performance of thin-film NC forward osmosis membranes. Compared to GO nanosheets, PVP-GO exhibited better dispersion and reduced aggregation tendencies. Furthermore, PVP-GO-modified forward osmosis (FO) membranes have demonstrated excellent desalination performance, including enhanced hydrophilicity, increased water flux, and reduced reverse solute flux compared to pristine and GO-modified FO membranes [56].

### 4.2. Chemical Techniques

Chemical surface modification is a powerful approach for tailoring the surface properties of NMs, ensuring their stability for various applications. These methods rely on forming covalent bonds between modifiers and the NM’s surface. Various coupling agents, including thiols, amines, organophosphorus compounds, carboxylic acids, polymers, and silanes, have been employed to enhance the dispersion of NMs in different media [49,57]. Wang et al. conducted surface treatments of fabrics coated with FeO, CoO, NiO, and CuO NPs using n-octadecyl thiol. Post-surface modification with thiol, the fabric/sponge, has exhibited remarkable superhydrophobic properties. The synthesized superhydrophobic/superoleophilic fabrics effectively separated both heavy and light oils [58]. Gheonea et al. described the functionalization of tin oxide (SnO_2_) surfaces using phosphonic acids, including phenyl-phosphonic acid and vinyl-phosphonic acid. They found that the surface treatment of SnO_2_ NPs with phenyl-phosphonic acid resulted in a higher modification yield compared to treatment with vinyl-phosphonic acid [59]. Sahoo et al. [60] introduced three different chemical functional groups, aminophenyl (C_6_H_4_NH_2_), nitrophenyl (C_6_H_4_NO_2_), and benzoic acid (C_6_H_4_COOH), to the sidewalls of MWCNTs to optimize their compatibility with liquid crystalline polymer (LCP). They examined how electrons donating and withdrawing groups attached to the functionalized MWCNTs affected their dispersion in the LCP matrix and their interaction with the LCP. The results indicated that the –C_6_H_4_NH_2_ functionalized MWCNTs exhibited the highest intermolecular interaction with the LCP, leading to significant changes in mechanical and rheological properties [60]. In 2017, Wu and colleagues produced nano-capsules using polylactic acid (PLA). However, PLA has certain drawbacks, such as brittleness, low impact strength, slow crystallization, and poor thermostability. To address these issues, they incorporated CNTs to enhance the toughness of PLA. Prior to preparing the PLA composite, they modified the CNT with methacryloxypropyltrimethoxysilane (MPS) to improve their dispersibility and compatibility. The resulting nano-capsules exhibited significantly improved tensile strength, elongation, and impact strength [61]. Sainz-Urruela et al. successfully obtained different GO samples that had been covalently functionalized with tannic acid through easy, cost-effective, and environmentally friendly processes. Grafting TA onto the GO surface enhanced solubility and dispersibility in organic solvents, improved thermal resistance, and increased antibacterial activity. Additionally, the modified samples exhibited significant increases in characteristic degradation temperatures (up to 60 °C) compared to pristine GO [62]. In the study conducted by Lyu et al. (2019) [63], a method was employed to synthesize a new composite material with desirable properties for enhanced oil recovery (EOR) applications in high-temperature and high-salinity reservoirs. The process involved covalently attaching vinyltriethoxysilane (VTES) molecules onto the surface of GO, resulting in the formation of surface-modified GO (sGO). Subsequently, sGO was copolymerized with acrylamide to produce the polyacrylamide, PAM/sGO, composite. The composite exhibited unique characteristics, including thermal resistance, shear stability, and tolerance to high salinity [63]. An overview of both modification techniques is shown in Table 1.

**Table 1:** Overview of surface modification methods for NMs.

Modification Method	Modifying Agent	NM	References
Physical technique	SDS	TiO_2_	[51]
	CTAB	Fe_3_O_4_	[52]
	Octylamine	CNT	[53]
	CTAB	CNT	[54]
	SDS	GO	[55]
	PVP	GO	[56]
Chemical technique	n-octadecyl thiol	FeO, CoO, NiO, CuO	[58]
	Phosphonic acid	SnO_2_	[59]
	Aminophenyl, nitrophenyl, benzoic acid	CNT	[60]
	MPS	CNT	[61]
	TA	GO	[62]
	VTES	GO	[63]

## 5. Chemistry of Silane

Silane, the fundamental component of silane chemistry, is a monomeric silicon (Si) compound with four substituents connected to it. Si belongs to the carbon family; therefore, it is the 14th group element in the periodic table with four valence electrons. Si has a vacant 3D orbital and, because of which, it shows distinct chemical and physical properties, such as a certain structure and reactivity that is unlike other members of the family [64,65]. Si also shows few characteristics that are similar to carbon, such as, for instance, catenation properties. Carbon can potentially create a lengthy chain with more carbon atoms (–C–C–)_n_ without limit. Likewise, Si can also build bonds with other Si atoms (–Si–Si–)_n_ but not extensive ones, perhaps because of the lower (Si–Si) bond energy. On the contrary, Si has the capability to build extended chains with oxygen and generate a siloxane linkage (–O–Si–O–)_n_ since the Si–O bond has quite a higher bond energy [66].

**Figure 2:**
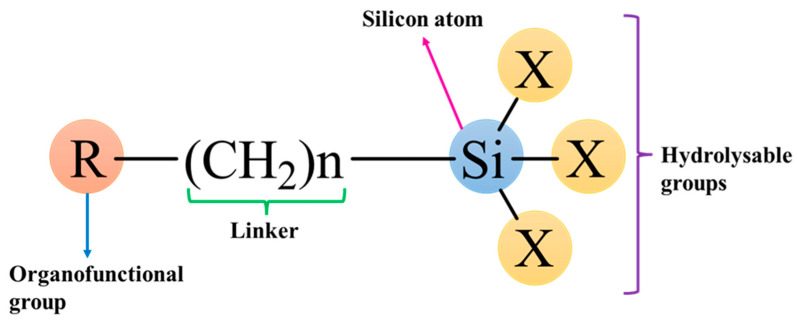
General formula of a silane coupling agent.

Silanes are literally referred to as SiH_4_, or they can be called silicon hydride, which is analogous to methane (CH_4_). The silanes that are largely used for modification purposes are organofunctional silanes. The organofunctional silane, or organosilane, is a silane with Si-C bonds. It typically consists of a silicon atom bonded to two types of reactive groups, one of which is the organofunctional group, including alkyl, aryl, or other organic moieties; and the other is the hydrolysable group, including the alkoxy group (as depicted in Figure 2). They have a basic structural formula of R_n_(CH_3_)_n_SiX_3_, where R can be an alkyl, aryl, or organofunctional group, and X can be a methoxy, ethoxy, or acetoxy group [67,68]. Organosilanes could interact with both inorganic and organic substances, between themselves, and also with other silanes via hydrolysis-condensation reactions to create a wide range of hybrid organic–inorganic structures; therefore, they can be used as adhesion promoters or coupling agents [69]. The alkoxy group present on organosilanes is able to react with inorganic materials, pigment, glass, steel, or filler to make a covalent bond. On the other hand, the organofunctional part can react with organic materials, resins, or polymers [70].

### 5.1. Classification of Silanes

**Figure 3:**
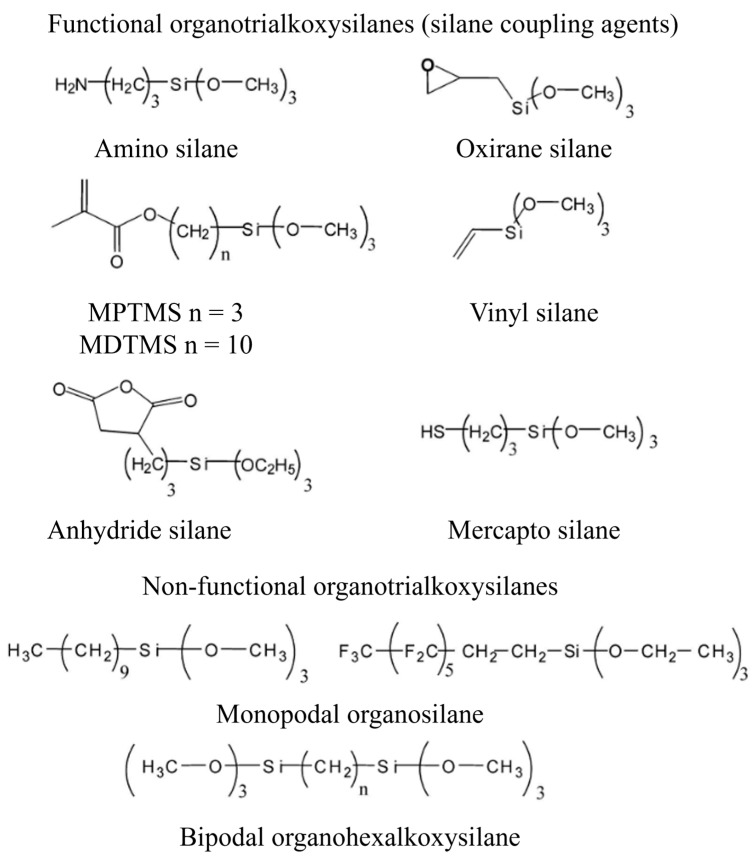
Functional and non-functional organo-alkoxysilanes (with silicon shown in its tetravalent state (valence = 4)) [65]. The figure was reproduced with permission.

Organosilane can be classified as functional and non-functional. Non-functional organosilanes have non-reactive R groups, such as hydrocarbon, fluorocarbon, etc. They usually have application as protective agents. Functional organosilanes have reactive dual functionalities, such as amino, mercapto, and vinyl groups, along with alkoxy groups; hence, they can be used as coupling agents. The examples of functional and non-functional silanes are depicted in Figure 3. Non-functional silanes can interconnect with functional silanes to form a cross-linking network that can lead to an increase in the density of the siloxane network. Hence, the amount of energy required to break the network bonding will be higher and, consequently, it will be hard for water molecules to diffuse into the siloxane network; thus, it obtains a higher hydrolytic stability in a water-containing environment. These crosslinker silanes can predominantly be used in dentistry [64]. Functional organosilanes can be monofunctional, difunctional, or trifunctional based on the number of reactive and hydrolysable groups, which are mostly alkoxy groups present on them. The monofunctional and difunctional organosilanes have one and two hydrolysable functionalities, respectively, while the trifunctional organosilanes have three. Most of the functional organosilanes that are used as coupling agents are generally trifunctional organosilanes. Organofunctional trialkoxysilanes, which are trifunctional organosilanes, were first introduced as a coupling agent in 1940 while fabricating fiber-glass-reinforced composites [71,72]. In addition, there are dipodal silanes that are another kind of organosilane, i.e., ones with two trifunctional silanes [69]. They can form a tighter network with the surface, providing a higher hydrolysis resistance.

### 5.2. Mechanism of Silane Modification

**Figure 4:**
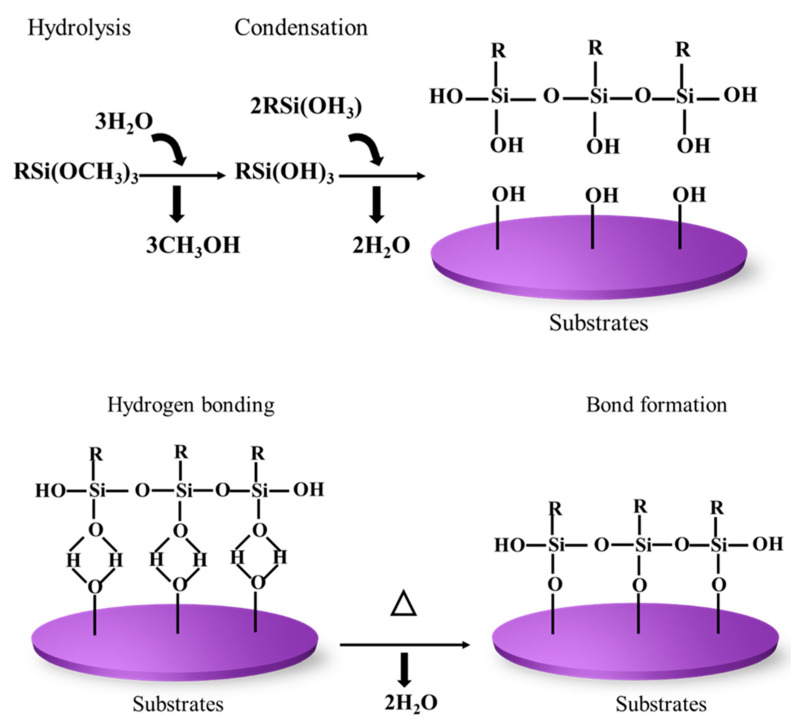
Silane modification mechanism.

Silanes are a hydrolysable group that can be hydrolyzed to silanol-carrying species. Mostly, trialkoxysilanes are used as coupling agents. The modification reaction essentially happens in four steps. The first step is the hydrolysis of the one alkoxy group in the presence of water or moisture. After that, the second and third alkoxy group hydrolyses and forms a silanol group, that is, the OR group is converted to Si–OH. The next step is the condensation of the silanols (Si–OH) to form siloxane linkage (Si–O–Si) by lateral bonding. The lateral bonding or crosslinking helps silane coating to be dense and compact, which helps it to improve its protecting effect [73]. Due to the greater electropositive character of Si, the dipole moment of the silanol group will be more, which leads to hydrogen bonding with OH on the substrate. Finally, the condensation of the OH group onto the surface of inorganic substrate occurs. All of the steps involved in the hydrolysis-condensation process happens simultaneously [67,72]. The mechanism of silane modification is shown in Figure 4.

Silane forms strong coupling with glass, silica, etc., due to its ability to form strong siloxane linkage while forming a mild coupling with substrates such as metal and metal alloys. Silanes can undergo hydrolysis more easily by acid than the basic condition, and they, subsequently, undergo condensation with the OH present on the substrate. In acidic conditions, initially, the alkoxy group is protonated. Following protonation, bimolecular nucleophilic substitution (S_N_2) happens where there is a backside attack of water molecules (H–O–H), which is a nucleophile on to the Si atom. This S_N_2 reaction leads to a pentacoordinate trigonal bipyramidal intermediate, and then a new bond will be formed between the water molecule and silicon atom. Simultaneously, a bond breaking will occur with the leaving alcohol, and then silanol will be formed. This step continues until all the alkoxy groups are replaced by a hydroxyl group and result in an inverted configuration product that is stable in pH around 3 [64,74]. Janamphansang et al. synthesized VO_2_ NPs and introduced APTES modification to mitigate particle aggregation. During the grafting process, the polar surfaces of VO_2_ adsorb water molecules, which then undergo hydrolysis with organosilane, generating hydroxysilane. The van der Waals forces between polar VO_2_ surfaces and the -OH groups of silanes result in some aggregation among hydroxysilanes. As they approach one another, condensation between Si-OH groups occurs, leading to the formation of a Si-O-Si crosslinked network with neighboring silanol groups. This Si-O bonding acts as a robust connection point between the anchoring group and the VO_2_ surface, ultimately forming a substantial coating layer on the MO surface. In this mechanism, the presence of water molecules plays a crucial role in the hydrolysis of ethoxy groups into silanol groups for subsequent condensation reactions [75].

Nylander and colleagues (2018) [76] used epoxy-silane monolayers, comprising glycidoxypropyltrimethoxysilane (GPTMS) molecules for covalently attaching CNT arrays to silicon substrates, which create thermal interface materials (TIMs). To enable covalent bonding, amino groups were incorporated into the CNT structure, providing the required chemical functionality to react with the epoxide groups on the silane monolayer. The thermal interface resistance of the resulting samples was measured using the laser flash technique, revealing a small but consistent improvement [76]. For usage in cementitious NCs, Silvestro et al. (2021) explored the modification of CNT via APTES because CNT has little interaction with the cementitious matrix. The hydrophilicity of the CNT can be decreased by silanization, which can increase the dispersion of the CNT in the cementitious matrix and, as a result, enhances their mechanical performance [77]. Surface-modified graphene/PVDF (SMG/PVDF) composites were made by Lin et al. (2019) utilizing APTES. It was discovered that as-synthesized SMG/PVDF composites showed significantly better dielectric properties compared with that of pristine PVDF, as well as RGO/PVDF composites, within the percolation threshold. This was due to the shielding effect provided by the PVDF coating layer over the graphene filler, as well as the proper dispersion of the SMG/PVDF within the PVDF matrix [78].

### 5.3. Factors Affecting Silane Modification

Silane modification depends on various factors, such as the bulkiness of alkoxy groups, pH, temperature, and solvent. The rate of hydrolysis is majorly affected by the bulkiness of the alkoxy group. The hydrolysis rate is usually lower for larger alkoxy groups in the order pentoxy < butoxy < propoxy < ethoxy < methoxy. This happens because there will be greater steric repulsion for the bulky alkoxy group [79].

The pH and temperature are also significant influencing factors in silane hydrolysis. In acidic and basic pH, there is an increase in the rate of hydrolysis, while, at a neutral pH, the hydrolysis rate reaches the minimum [64]. This happens because, in acidic and basic pH, there is abundant H^+^ and OH^−^ ions that can accelerate silane hydrolysis by acting as a catalyst [64]. Temperature is another crucial factor. The rate of the hydrolysis reaction rises with temperature in accordance with the Arrhenius law [80].

Silane hydrolysis also relies on the nature of solvent. As the hydrophilicity of solvent decreases in the order of methanol, ethanol, and propan-1-ol, the hydrolysis rate also decreases. This is attributed to the capability of isolating the “free” water molecule from the bulk water state. Usually, ethanol is used as the solvent as methanol is toxic [81].

### 5.4. Silanes Used for the Modification of NMs

**Figure 5:**
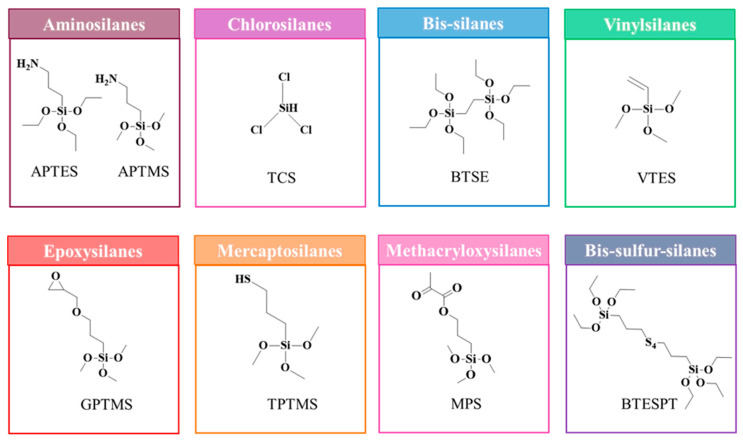
Various silane coupling agents for modifying NMs.

The surface modification using silanes on any surface reaches its maximum when they react with the surface more efficiently and provide a greater number of attainable sites that have proper surface energies. Regarding NM silanization, APTES and 3-aminopropyltrimethoxysilane (APTMS) have been widely used as silane agents. The electron-rich nitrogen atom of the amine group easily interacts with hydrogen groups, such as hydroxyl groups on the NM surface or other amines [77]. Sun et al. (2016) [82] prepared MWCNTs functionalized with amino groups via silane treatment. By covalently connecting an amino-terminal silane (APTMS) into activated CNT, they chemically altered MWCNTs. With the employment of the silane-coupling technique, the amino-terminal MWCNTs (AMWCNTs) retained the carbon framework structure, even after grafting additional functional groups. The presence of amino groups significantly enhanced the hydrophilicity of MWCNTs, as evidenced by the fact that the as-prepared AMWCNTs were evenly distributed in water and showed no aggregation over many weeks. These AMWCNTs were utilized in the creation of glucose biosensors that enable the detection of glucose [82]. Mishra et al. (2019) [83] evaluated the impact of chemical modification and dispersion of ball-milled CNT-integrated epoxy resin. When compared to neat resin, silane-modified CNT/epoxy resin exhibited an improvement in fracture toughness by 38% and tensile modulus by 42%. The enhancement of mechanical properties in silane-modified CNT/epoxy composites has been attributed to the strong adhesion between the CNT-matrix interface, which is facilitated by the presence of silane modification. The silane treatment also promoted efficient dispersion of the CNT within the epoxy resin [83]. Huang et al. (2022) [84] conducted a study where they made modifications to nano-Al_2_O_3_ particles and GO nanosheets using APTES. These modified particles were then dispersed in epoxy resin, resulting in the creation of modified-Al_2_O_3_/epoxy, modified-GO/epoxy, and modified-Al_2_O_3_@GO/epoxy composite coatings on steel sheets. The addition of a small quantity of NPs led to an excellent dispersion of the particles within the epoxy resin. Notably, when the content of the nano-Al_2_O_3_ particles reached 1.5 wt.%, the particles exhibited the most favorable dispersion and stability within the epoxy. The incorporation of these NPs significantly enhanced the hardness, abrasion resistance, and corrosion resistance of the composite coatings [84]. Many researchers have employed various silane agents to modify NMs, including MPS, VTES, GPTMS, bis-(triethoxysilylpropyl)-tetrasulfide (TESPT), triethoxy(octyl)silane (TEOS), etc. The structure of various silanes is given in Figure 5. Jiang et al. (2020) [85] conducted research on the development of a thermosetting resin system utilizing bismaleimide (BMI) as a base, which was enhanced with a newly synthesized GO modified using GPTMS, an epoxy silane (ES-GO). Through thermogravimetric analysis, it was determined that the cured sample systems exhibited a significant increase in char yield at lower concentrations of ES-GO (≤0.5 wt.%), indicating an enhanced thermal stability. Additionally, dynamic mechanical analysis (DMA) revealed a notable rise in the glass transition temperature (T_g_) as the content of ES-GO increased [85]. The impact of silane-modified graphene nanosheets (SMGNs) upon the physical and mechanical characteristics of vinyl–ester resin composites was studied by Gholiha et al. (2020) [86]. The surface of graphene was modified using VTES. It was discovered that the interface within the nanosheets and vinyl–ester matrix was enhanced by the silane-functionalized NCs. Additionally, it was discovered that surface modification might greatly enhance GNS’s adhesion and dispersion compared to clean vinyl–ester with unmodified composites [86].

## 6. Silane Modification of NMs

### 6.1. Fullerene

The fullerene family is unique in both physical and chemical characteristics. The very stable chemical Buckminsterfullerene (C_60_) was originally discovered in 1985 [87]. After diamond and graphite, the fullerene family is regarded as the third allotrope of carbon. Several fullerenes, some in several isomeric forms, have now been identified, including chiral and achiral varieties, like C_70_, C_76_, C_78_, C_82_, C_84_, etc. [88]. There are additional large fullerenes with an n of 240, 560, and 960, which group together in quasi-icosahedral patterns [89]. The photophysical and medicinal uses of functionalized C_60_ molecules are the two most prominent of the many possible application fields [90]. A variety of C_60_-based systems have been created and employed as organic solar cells due to the C_60_ cage’s exceptional electron acceptor characteristics. In order to safeguard optical sensors against excessive laser light, C_60_ also functions as an optical limiter. The most significant prospective medicinal uses for C_60_ compounds are based on their displayed antiviral activity, their use in the role of photosensitizer in photodynamic treatment, and their behavior as an antioxidant due to their capacity to scavenge free radicals [91]. To make fullerene more compatible with different materials and to give it better application-specific qualities, it is possible to modify the surface of fullerenes by covalently bonding silane molecules to the surface on the fullerene structure [92]. Fullerenes’ electrical characteristics and energy levels may also be changed by silane modification, which allows for fine tuning of their performance within electronic, as well as optoelectronic, devices [92]. The enhanced solubility of derivatives of fullerene into organic solvents or to polymers is a key advantage of silane modification. Due to their improved solubility, fullerenes may be processed more easily and integrated into a variety of device topologies [93].

**Figure 6:**
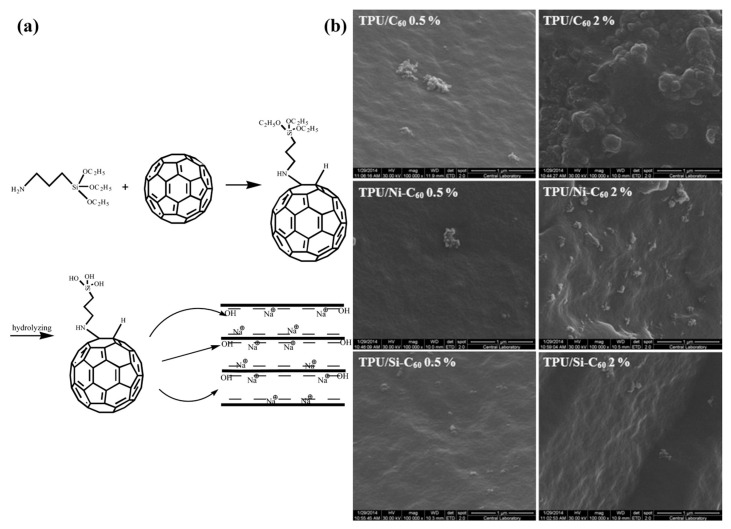
(**a**) Diagrammatic representation of the synthetic pathway for C_60_-decorated MMT (C_60_-Si-MMT) [94]. (**b**) SEM images of the pristine and surface-modified fullerenes [92]. All figures were reproduced with permission.

Chen et al. (2021) [94] employed the silane coupling agent APTES to connect montmorillonite (MMT) with fullerene (C_60_) in order to synthesize a hybrid material known as C_60_-decorated MMT (C_60_-Si-MMT). Subsequently, composites of polypropylene (PP) with a loading of 3.0% by mass of C_60_-Si-MMT were prepared through melt compounding. Thermal gravimetric (TG) and differential scanning calorimetry analyses revealed that the inclusion of C_60_-Si-MMT significantly enhanced the onset degradation temperature (T_onset_) and the maximum decomposition temperature (T_max_) of PP to 287.9 °C and 394.7 °C, respectively. Rheological investigations, along with TG analysis coupled with Fourier transform infrared spectroscopy (TG-IR), have demonstrated that the well-dispersed C_60_-Si-MMT within the matrix effectively trap the free radicals generated by C_60_, while MMT effectively restricts the segmental movement of the macromolecular chain. The schematic synthetic route for C_60_-decorated MMT (C_60_-Si-MMT) is depicted in Figure 6a [94]. Das et al. (2016) [95] conducted a study where pristine C_60_ was chemically modified by oxidation in the presence of nitric acid, and this was followed by silanization using APTES, resulting in the introduction of oxygenated and siloxane functional groups onto C_60_ surfaces. Epoxy NCs were then fabricated by incorporating 0.5 wt.% of both pristine and stepwise modified C_60_. The impact of C_60_ surface modification on the properties of the epoxy NC was investigated using DMA and TGA techniques. The results revealed that the silanized C_60_/epoxy NC exhibited a substantial improvement in the storage modulus compared to the neat epoxy. TGA analysis showed a modest increase in the initial decomposition temperature for the silanized C_60_/epoxy NC. Overall, these findings demonstrate the beneficial effects of the surface modification of C_60_ on the properties of epoxy NCs [95]. In another study, Das et al. (2018) utilized oxidized and silanized fullerene (C_60_) to develop a high-performance epoxy NC with improved anti-scratch properties. Oxidation of C_60_ led to the formation of interconnected chainlike networks, which were subsequently concealed after silanization using APTES. The silanized C_60_ exhibited active functional groups that regulated polymer assembly and coordination, resulting in a significant enhancement in the scratch recovery index (~32%), wear resistance (~57%), and micro-hardness (~19%) of the epoxy composites. Furthermore, the incorporation of silanized C_60_ as a nano-reinforcement in epoxy provided a dual advantage by significantly improving the mechanical properties. The tensile strength, elastic modulus, and fracture toughness of the epoxy NCs were enhanced by ~28%, ~30%, and ~67.5%, respectively. This work highlights the potential of silanized C_60_ for enhancing the performance of epoxy-based materials [96]. Tayfun et al. (2015) [92] conducted a study on functionalizing fullerene using nitric acid and the amino-functional silane coupling agent, APTES. The pristine and surface-modified fullerenes were then incorporated into a TPU matrix through the melt mixing method. The surface modifications of the fullerene led to a significant improvement in the tensile strength and percent elongation of the composites, approximately doubling these properties compared to TPU alone. This enhancement was attributed to the improved dispersion resulting from the enhanced interactions between the fullerene and the TPU matrix. Figure 6b shows the results of the scanning electron microscopy (SEM) analysis, which demonstrated a gradual reduction in the formation of agglomerates and a more homogeneous dispersion of fullerene particles following the surface modifications [92].

### 6.2. CNT

CNT is one of the most important nano-material invented in 1991 by Lijima and Ichihashi. CNTs have fueled the era of nanotechnology [97,98]. As already mentioned, they are one-dimensional nano-materials also known by the name bucky tube. They are formed by rolling up graphene sheets. There are two kinds of CNTs, single-walled CNTs (SWCNTs) and MWCNTs [99]. The SWCNT is twisted single graphene layers, while the MWCNT is composed of two or more graphene layers packed by van der Waals forces [100]. CNTs have gained great attention owing to their exceptional properties, like biocompatibility, fast electron transport, mechanical and thermal stability, and high surface reactivity with biomolecules. CNT has commonly been used in thermal conductors, energy storage materials, conductive adhesives, thermally stable materials, structural materials, fibers, catalyst supports, biological applications, air and water filtration, ceramics, and other applications [101]. Though there are advantages to CNT, they are also certain drawbacks. Like all NMs, because of the existence of the strong van der Waals forces among CNTs, they tend to agglomerate and reach the lowest energy state possible, resulting in a non-uniform dispersion in the medium. As discussed earlier, silane modification can help in overcoming issues. The silane modification of CNTs is a well-established technique and has been reported in the literature for more than 20 years.

**Figure 7:**
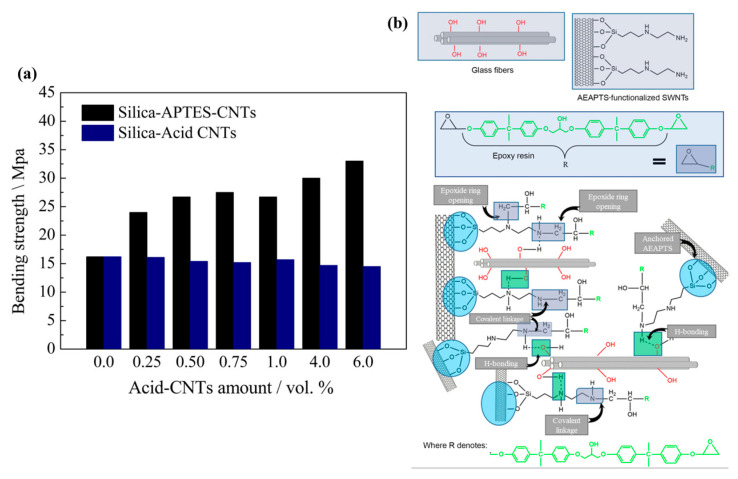
(**a**) Bending strength assessment of silica-acid CNTs and silica-APTES-CNTs with different CNT vol% [110]. (**b**) An illustration depicting the chemical interactions between AEAPTS-functionalized SWCNTs, epoxy, and glass fibers [112]. All figures were reproduced with permission.

Gulati et al. (2018) developed an optical biosensor utilizing CNTs as electro-optic material in order to identify chronic myeloid leukemia (CML), and this was performed because CNT has the ability to detect trace amounts of certain biomolecules via photo-induced electron transfer. With the help of GPTMS molecules, the CNT surface was modified, which allows for covalent immobilization with certain antibody molecules and direct intermolecular reactions, giving significant stability, as well as selectivity, for the sensor [102]. CNT must undergo oxidation to insert carboxyl (COOH) and hydroxyl (OH) groups on their surface because silanes bind to CNT through these functional groups. The proper dispersion of CNT in a polymer was investigated by Kalyani et al. (2020) [103] using modification, silanization, treatment with amines, etc. They compared the characteristics of CNTs that had been carboxylated, silanized, and thiolated, as well as aminated. Silane-treated MWCNTs have improved mechanical characteristics. CNTs adhere well to the polymer matrix in MWCNTS modified with silane and additional functionalization [103]. The silanization of CNT has significant influence on their mechanical, physical, and electrical properties in NCs. The effect of functionalization and silanization on the mechanical properties of polymer-based/(SWCNT) NCs with PP and polyvinyl chloride (PVC) as the selected polymers was investigated by Mashhadzadeh et al. (2017) [104]. The silanization of MWCNT with APTES enhanced the chemical adaptability of MWCNT significantly, and it also heightened the adsorption energy and tensile and flexural properties of the silanized-CNT/PP and silanized-CNT/PVC [104]. Silane-functionalized CNT has an influence on the physical and electrical characteristics of linear low-density polyethylene (LLDPE)/MWCNT NCs, which was examined by Azizi et al. (2020) [105]. VTES was used to modify CNTs, which were then added as a nanofiller to create a NC, and this helped in boosting the mechanical and electrical properties of the NC [105]. For integrating CNTs in a natural rubber (NR) matrix, Ding et al. (2017) modified them using a silane coupling agent TESPT to create functionalized CNTs (F-CNTs). It resulted in a strong and flexible network with improved dispersion of the F-CNTs in the NR matrix. From crack propagation tests, they found that the F-CNTs exhibited better crack resistance than the unmodified CNTs [106]. For application in wearable accessories, Hong et al. (2020) [107] produced a novel conductive fiber by combining CNT into a polyurethane (PU) matrix. In order to maintain the mechanical traits, CNT was coated with (heptadecafluoro–1,1,2,2–tetra–hydrodecyl)trichlorosilane prior to adding it into a PU that was connected to the composite with a strong network. Furthermore, it increased the fiber’s strain value [107]. In another paper, the MWCNTs were chemically immobilized on a glass surface by Takada et al. (2020) [108] utilizing a silane coupling agent p-(chloromethyl) phenyltrimethoxysilane (p-CMPTMS) that was photoreactive, and this was then hydrolyzed with acetic acid. The MWCNT-coated glass can be used as self-heating material for the windows of automobiles used in cold climates and as a panel for measuring devices used in low-temperature settings [108]. Goriparthi et al. (2019) [109] used CNTs to reinforce polyoxymethylene (POM) to create POM/CNT composites, which allowed them to study the mechanical, wear, and fatigue characteristics of POM. Several functionalization techniques, including carboxylation, silanization, carbonylation, and amination, were used to improve the interaction between CNTs and POM. They silanized the CNT surface using APTES to give S-CNTs, which showed better dispersion, as well as physical and chemical properties, than the neat CNT NCs [109]. Peng et al. (2018) [110] produced a composite material composed of silica (SiO_2_) NPs and CNT by sintering. However, sintering CNT-based ceramic composites is difficult; hence, to address this challenge, they added a silane coupling agent called APTES, which can bond to both silicon atoms and CNTs. Functionalizing CNTs with APTES improves their dispersibility and reduces agglomeration. Additionally, they also discovered that silica-APTES-CNT composites exhibited stronger bending strength with all proportions than silica-acid CNTs composites, as shown in Figure 7a [110]. In the Kang et al. (2020) [111] study, they produced NCs of polyamide 66 (PA66) with dispersed MWCNTs (PA66/MWCNTs) through melt-mixing previously treated MWCNTs and PA66. The pre-treatment materials were treated by an epoxy modifier, an ethoxylated non-ionic fluoro-surfactant, and a silane coupling agent APTES, among which silane exhibited the greatest improvement in physical characteristics, like a 71.1% rise in the Young’s modulus and a 47.6% hike in tensile strength [111]. In order to improve the thermomechanical properties of the glass fiber/epoxy (GF/epoxy) composites, Jamshaid et al. in 2020 [112] looked into the possibility of using functionalized SWCNT (f-SWCNTs) that had been functionalized with N-(2-aminoethyl)-3 aminopropyl trimethoxysilane (AEAPTS) as an interface modifier. Through mechanical testing, they revealed that, if f-SWCNTs were present, the dispersion and wetting of the glass fibers demonstrated a high degree of compatibility with the epoxy resin, resulting in better interfacial adhesion; hence, AEAPTS functions well as an interphase modifier. An illustration depicting the chemical interactions between AEAPTS-functionalized SWCNTs, epoxy, and glass fibers is given in Figure 7b [112]. Lu et al. (2017) [113] developed a method to create super-hydrophobic/superoleophilic ethyl cellulose (EC) sponges by cross-linking with epichlorohydrin (ECH) and complexing with silanized CNTs (Si-CNTs). The surfaces of the CNTs were silanized with hexadecyltrimethoxysilane (HDTMS) to improve the compatibility of the CNTs with EC, thereby increasing the hydrophobicity. The sponges exhibited high porosity, excellent oil absorption properties, exceptional recyclability, and durability, making them ideal absorbents for cleaning up oil spills [113]. The impact of surface-treated MWCNTs on the compressive characteristics of unidirectional (UD) kenaf and hybrid woven glass/UD kenaf fiber-reinforced polymer composites was studied by Sapiai et al. (2020) [114]. To enhance the dispersion of MWCNTs inside the epoxy matrix, they treated the MWCNTs with a solution of H_2_SO_4_ and HNO_3_, as well as with APTES. The results showed that the inclusion of processed MWCNTs improved the compressive characteristics of kenaf, as well as hybrid composites, particularly with silane treatment, which compensated for the poor compressive performance of kenaf composites [114]. Mozaffarinasab et al. (2023) [115] utilized a Bis(3-triethoxysilylpropyl)amine (BTESPA) for the surface modification of MWCNTs. Both pristine and modified nanotubes were incorporated into epoxy resin at various concentrations (0.5, 1, and 2 wt.%). The effects on mechanical properties, curing behavior, viscoelastic properties, thermal behavior, and morphology of the pure and modified MWCNT-containing epoxies were investigated. The findings revealed that incorporating pristine nanotubes at a concentration of 0.5 wt.% enhanced the mechanical strengths of the epoxy sample. On the other hand, incorporating 0.5 wt.% of modified MWCNTs into the epoxy binder led to a significant improvement of 33%, 53%, and 40% in tensile, flexural, and compressive strengths, respectively, as well as an increment of 56%, 85%, and 110% in the tensile, flexural, and compressive modulus compared to the NC-containing pristine MWCNTs. Furthermore, the modified MWCNTs contributed to the curing of the epoxy resin, which resulted in an increase in the enthalpy of the curing by up to 25% [115].

### 6.3. Graphene

Graphene is a 2D NM with a hexagonal lattice arrangement and has a thickness in the nanometer range. It has been widely regarded as the thinnest, most durable, and most conductible material ever created [116]. Andre Geim and Konstantin Novoselov in 2004 isolated graphene and characterized it for the first time [117]. They received the Nobel Prize in Physics for this wonderful discovery. Graphene is composed of one or multiple layers of carbon atoms that are one atom thick, with each carbon atom sp^2^ hybridized and arranged in six-membered rings. Graphene is generally synthesized by Hummers’ method. The process of producing graphene using Hummers’ method involves the oxidation of graphite with various oxidizing agents, including sulfuric acid, sodium nitrate, and potassium permanganate. This is followed by the exfoliation of graphene oxide and reduction to obtain graphene [118]. Graphene possesses distinctive characteristics, such as exceptional tensile strength, Young’s modulus, electrical conductivity, thermal conductivity, barrier properties, chemical stability, thermal stability, and remarkable lubricating properties, which has seen it, consequently, attract a great deal of attention [119,120,121]. Graphene has a broad range of applications, such as in semiconductors, battery electrodes, capacitors, electrocatalysis, corrosion resistance, 3D printing, gas separations, and wastewater treatment [120,122]. Compared to SWCNTs, graphene offers a greater surface area that makes it a good material for sensors, as well as catalysis. Furthermore, sorption can occur on both sides of the planar graphene sheet since both surfaces are accessible [100]. Other than all these advantages, graphene has drawbacks that obstruct its compatibility with organic materials: the strong van der Waals forces and p-p interactions between graphene sheets can cause aggregation and agglomeration [120]. The silane modification of graphene can help in preventing agglomeration. Here, the graphene can be functionalized with silane molecules. The silane modification can improve its dispersibility in solvents and enhance its compatibility with other materials. This is achieved by attaching the silane molecules to the graphene surface through covalent bonding or non-covalent interactions, which can alter the surface chemistry and properties of graphene.

**Figure 8:**
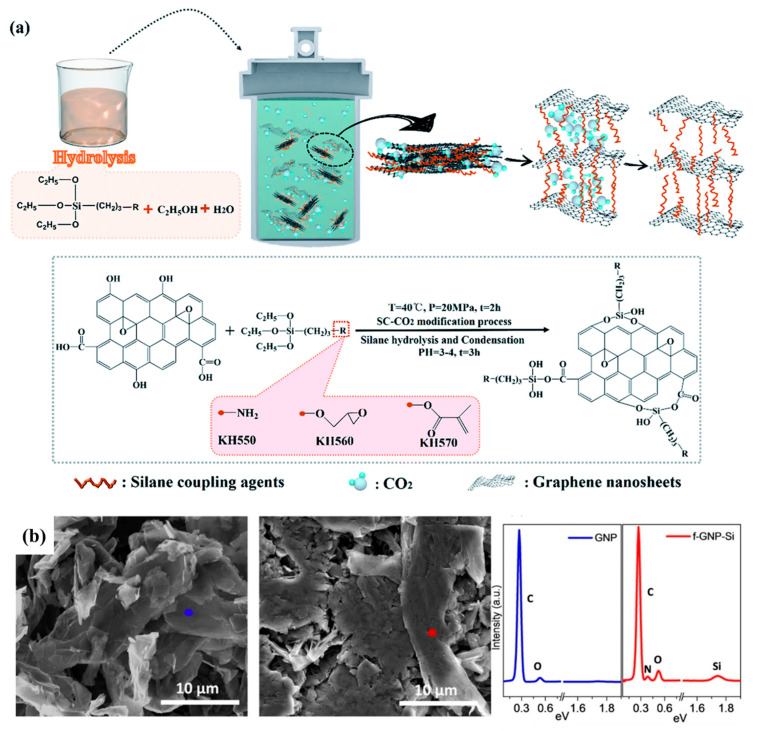
(**a**) A schematic illustration outlining the surface modification of GNs [124]. (**b**) The SEM images of GNP and f-GNP-Si, as well as the EDS single spectra of points, are marked in blue and (f-GNP-Si, respectively [126]. All figures were reproduced with permission.

Kang et al. (2022) [123] polymerized N-isopropylacrylamide (NIPAM) along with carboxymethyl chitosan (CS) to create a temperature-sensitive antibacterial hydrogel. To improve the mechanical characteristics of the hydrogel, NIPAM was additionally polymerized simultaneously over highly stable dispersed silane-functionalized graphene (GM). Graphene was coupled with MPS. This highly distributed graphene derivative, that is, GM, increased the hydrogel’s mechanical characteristics by adding additional crosslinking sites [123]. The influence of various silanes on the oxygen and water vapor barrier properties of graphene nanoplatelets (GNs) was studied by Wei et al. (2019) [124]. They developed a straightforward and environmentally friendly approach to modify GNs using various silane coupling agents, including APTES (KH550), GPTMS (KH560), and MPS (KH570). The modified GNs were then blended with polyethylene terephthalate (PET) to give GN550/PET, GN560/PET, and GN570/PET composite films (Figure 8a). Based on DMA results, it was observed that the interfacial interaction in GNs550/PET and GNs560/PET composites was notably stronger compared to the GNs570/PET composite. This difference can be attributed to the ability of the amino group in KH550 and the epoxy group in KH560 to interact with the terminal carboxyl and hydroxyl groups on the PET chain. In contrast, the terminal methyl group and carbon–carbon double bond in KH570 exhibited limited reactivity. They found that the GN550/PET and GN560/PET greatly improved the oxygen and water vapor barrier properties of the composite films, as determined through barrier property testing [124]. In another study, Guo et al. (2023) [125] employed the hydrothermal synthesis method to produce autoclaved wall blocks (SW-AWBs). To enhance the impermeability and freeze–thaw resistance of the SW-AWBs, they developed composite coatings by preparing MPS-modified nano-Al_2_O_3_ (NA) and graphene nanoplatelets (GNPs), which were mixed with TEOS. The agglomeration of NA and GNPs significantly diminished following the modification process. This indicates the effective dispersion of the modified NA and GNPs in TOS. As a result, there was significant enhancement in the freeze–thaw durability of the blocks, making them suitable for use as wall materials in cold regions [125]. Ren et al. (2022) [126] investigated the impact of incorporating silane-functionalized graphene nanoplatelets (f-GNP-Si) on reinforced silicone rubber (SR). The functionalization procedure, which involved APTMS, resulted in an increased surface roughness of the GNP flakes, as seen in the SEM images presented in Figure 8b. The improved dispersion of f-GNP-Si in the SR matrix was likely due to better compatibility and interfacial bonding. The mechanical properties of the f-GNP-Si SR composites were found to increase with increasing, up to 2 phr, the filler loading. The thermal conductivity of the composites also increased with increasing the f-GNP-Si loading, showing a significant enhancement of up to 150% at 5 phr [126]. Huang et al. (2023) [127] synthesized a graphene nanohybrid (D-GR) by grafting APTES onto a graphene surface. The incorporation of D-GR resulted in simultaneous enhancements in flame retardancy, mechanical performance, and thermal properties of epoxy resin. Specifically, a composition of 1 wt.% D-GR/EP achieved a vertical burning V-1 rating, with limiting oxygen indexes reaching 30.1%. Additionally, the presence of D-GR resulted in a 31.70% reduction in the discharge of toxic gases (CO) and an increase in the content of char residue [127].

### 6.4. Graphene Oxide

**Figure 9:**
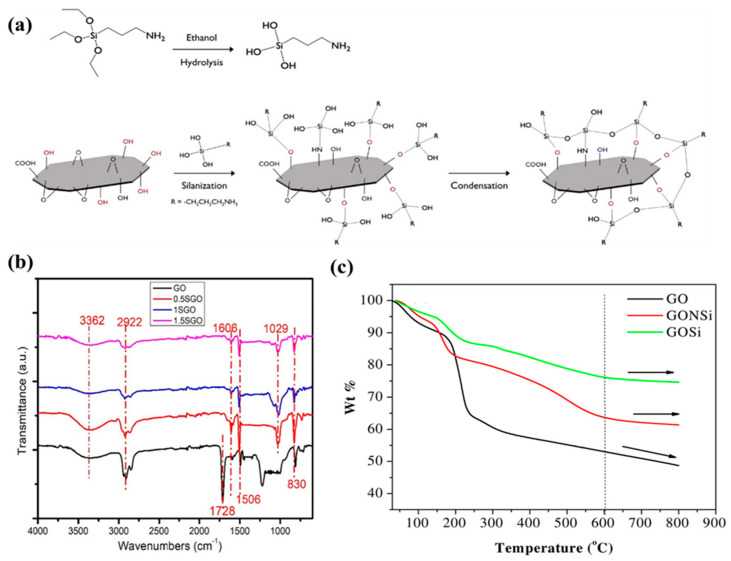
(**a**) Synthesis process of fGO–PU NCs [131]. (**b**) FTIR spectra curve of GO and SGO [135]. (**c**) TGA curve of functionalized GO compared to GO [137]. All figures were reproduced with permission.

Graphene oxide (GO) is a form of graphene that has oxygen-containing functional groups, such as hydroxyl, epoxy, and carboxyl, on its surface. It is typically produced by the oxidation of graphite, which results in the formation of layers of GO stacked on top of each other [128]. Due to its special characteristics, as well as potential uses, GO has gained considerable interest across a number of fields. Its high surface area, exceptional dispersibility in water, as well as other solvents, and the capacity to create stable colloidal suspensions, are a few of its key features. In addition, it has adjustable optical and electrical, as well as mechanical, properties owing to the extent of oxidation along with functionalization [129,130]. Similar to graphene, GO also has the same tendency to agglomerate. Silane modification of GO is one way to prevent agglomeration. Some examples of silanes used for modification include trimethylsilane, triethoxysilane, and APTES [126]. These silanes can react with the oxygen-containing functional groups on the graphene and GO surface, such as hydroxyl and carboxyl groups, to form stable covalent bonds. Lee et al. (2020) [131] synthesized functionalized graphene GO (fGO), wherein the GO was grafted with APTES. Subsequently, the fGO was incorporated into a polyurethane (PU) matrix. The preparation of fGO–PU NC is shown in Figure 9a. Various fGO–PU NCs were prepared with different concentrations of fGO. Remarkably, the kinetic analysis of activation energy (E_a_) revealed that even a minute addition of fGO resulted in an enhancement of thermal stability in the NCs. Moreover, all the fGO–PU NCs exhibited improved mechanical properties, such as increased tensile strength, elongation, toughness, and hysteresis, as the loading level of fGO was increased [131]. In order to enhance the interaction of GO with the polymeric matrix in a molten state, Torres Castillo and colleagues (2023) [132] functionalized it with APTES. The NCs of nylon 6,6 were then produced using a melt blending technique with both unfunctionalized and functionalized GO. They observed an increase in the crystallinity and glass transition temperature of the composite materials. Additionally, the composite samples containing functionalized GO (GOAPTS) exhibited higher values of loss modulus compared to those with unfunctionalized GO. The composite sample with a content of 0.6 wt.% of GOAPTS demonstrated the highest storage modulus [132]. The effect of amino-modified GO (AMG) in a NC consisting of AMG and polyamide 6 (PA6) was conducted by Chen et al. (2018) [133]. They introduced modifications to GO by anchoring N-(2-aminoethyl)-3-aminopropyltrimethoxysilane (AAPS) onto the surfaces of the GO sheets. The results demonstrated that the AMG exhibited excellent dispersity and enhanced thermal stability. The incorporation of AMG into the polymer composites led to significant improvements in their friction coefficient and wear resistance. Specifically, the friction coefficient of the AMG/PA6 composite was measured to be 0.237, representing a remarkable reduction of 44.6% compared to neat PA6. Additionally, the wear rate of the NC decreased by 33.2% [133]. Silane modification can lead to change in the mechanical and electrical properties of GO. Fazil et al. (2019) [134] modified GO by incorporating ethyltriethoxysilane (ETEOS), resulting in ETEOS-functionalized GO (EGO). This EGO was then utilized to create a composite material with polyimide (PI). The mechanical properties of the NC demonstrated significant enhancement, with the addition of 0.2 wt.% EGO leading to a remarkable 56% increase in the Young’s modulus compared to PI alone. Moreover, the NC exhibited improved dielectric stability, as evidenced by a decrease in the dielectric constant from 12.1 to 11.7 as the frequency increased from 1 kHz to 1 MHz. Additionally, the dissipation factor remained below 0.13, indicating promising characteristics for this dielectric-stable NC [134]. The properties of carbon fiber were modified by applying a coating of APTES-functionalized GO (SGO), as reported by Manu et al. (2022) [135]. The process involved depositing SGO onto a carbon fiber surface made of polyacrylonitrile (PAN)-based plain weave. To create carbon fiber-reinforced polymer (CFRP) laminates, the SGO-coated carbon fiber was combined with epoxy resin. Mechanical testing of the CFRP samples revealed significant improvements in the tensile, flexural, and impact strength at room temperature, with enhancements of 13.1%, 26.6%, and 3.9%, respectively. At cryogenic temperatures, the improvements were even more pronounced, with increases of 15.1%, 35.3%, and 13.5%, respectively. FTIR studies have confirmed the presence of a flexible Si-O-Si bond, which has been identified as a crucial factor contributing to the improved strength. The appearance of deflection peaks at 1029 cm^−1^ and 950 cm^−1^ in the FTIR spectra indicated the presence of “-Si-O-Si-” and Si-O-C groups on the SGO-coated surface, as shown in Figure 9b [135]. In another study, Tang and colleagues (2023) conducted a study in which they functionalized the surface of GO with APTMS (APTS). The researchers aimed to investigate the potential enhancement effects of this modified GO composite on the geopolymer system. The inclusion of GO-APTS led to significant improvements in the porosity and pore size distribution of the geopolymer matrix. Moreover, the functionalization of GO with APTS resulted in the enhanced flowability of the geopolymer. Notably, the incorporation of GO-APTS substantially improved the mechanical strength of the geopolymer, particularly in terms of tensile strength. Furthermore, the GO-APTS samples exhibited lower water permeability, as the total water absorption was approximately 30% lower compared to the control sample [136]. Rehim et al. (2019) [137] conducted a study to enhance the dispersion of GO in epoxy resin by functionalizing the GO with small silane, APTES (GONSi), and bulky silane moieties TEOS (GOSi). The incorporation of these modified forms of GO resulted in an improved thermal stability of the loaded epoxy resin. Additionally, investigations on the dielectric properties showed that the insulation feature of CE was not significantly compromised by the addition of GO or its modified versions. Thermal gravimetric analysis indicated that the loaded samples exhibited higher thermal stability compared to the pure resin, with the T10% increasing from 273 to 324 °C for GONSi (1 wt.%), despite the low loading content (Figure 9c). This approach effectively enhanced the dispersion of the GO in epoxy resin while maintaining its desirable thermal and dielectric properties [137]. Jamali et al. (2019) [138] explored the effects of silane-functionalized graphene oxide (GO) nanoplatelets on basalt fiber (BF)/epoxy composites’ mechanical properties. They initiated the study by organically modifying GO with N-(3-trimethoxysilylpropyl)ethylenediamine (3-TMSPED). SEM analysis revealed that the silanized-GO (SGO)/BF/epoxy composite displayed markedly improved interfacial bonding between BF and the epoxy matrix compared to the non-silanized GO composite. SGO demonstrated superior effectiveness in enhancing the mechanical properties of fibrous composites, and these were attributed to its silane functionalization [138]. Further advancement was made by Jamali et al. (2021) [139] by developing basalt fiber/epoxy composites reinforced with GPTMS-silanized GO, with the optimal loading determined as 0.4 wt.%. This resulted in significant enhancements in tribological properties, including a 62% reduction in wear rate and a 44% decrease in the friction coefficient. Additionally, the composite containing 0.4 wt.% silanized-GO exhibited the highest improvements in the storage modulus (130%) and glass transition temperature (Tg, 13.6 °C) compared to the neat composite [139].

### 6.5. Metal Oxides

MO NMs are constituted of metal atoms and oxygen atoms. They have unique properties due to their small size, which makes them useful in various applications, such as catalysis, sensors, energy storage, and biomedical applications [140,141]. Some examples of MO NMs include copper oxide (CuO), zinc oxide (ZnO), titanium dioxide (TiO_2_), iron oxide (Fe_2_O_3_), and aluminum oxide (Al_2_O_3_) [142]. These materials have a high surface area-to-volume ratio, which enhances their reactivity and allows them to interact more effectively with their surroundings. MO NMs can be synthesized using various techniques such as sol–gel synthesis, hydrothermal synthesis, and vapor-phase deposition [143]. Silane modification is a widely used method to functionalize MO NMs. This method involves the reaction of silanes, such as alkoxysilanes or chlorosilanes, with the surface hydroxyl groups on the MO NMs. These can improve the dispersibility, stability, and reactivity of the NMs in various environments, such as in aqueous or organic solvents, or in different matrices, such as polymer composites. APTES-mediated surface modification of ZnO NPs was first reported by Grasset et al. as far back as 2003 [144].

**Figure 10:**
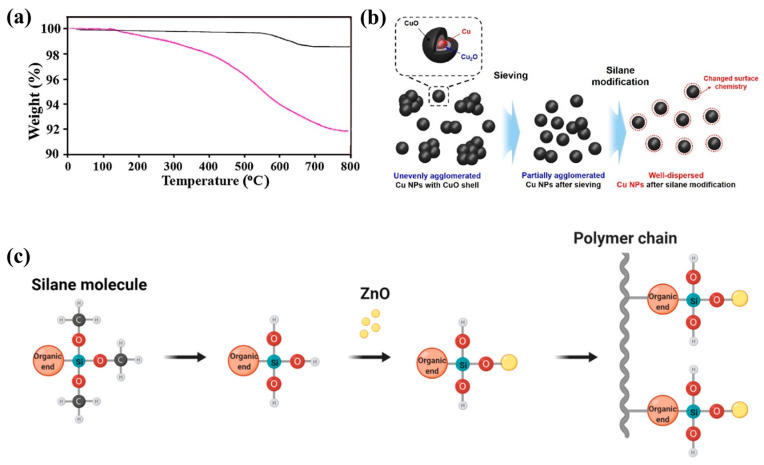
(**a**) TGA thermograms of pristine TiO_2_ and surface-modified TiO_2_ [146]. (**b**) Diagram illustrating the dispersion process achieved through surface modification using a silane dispersant [148]. (**c**) Schematic diagram depicting the modification process of ZnO with MPTMS [151]. All figures were reproduced with permission.

Ma et al. (2022) [145] utilized the silane coupling agents’ TEOS and APTES to modify the surface of ZnO NPs via covalent coating. This surface modification technique effectively enhances the UV blocking capabilities of ZnO NPs, improves their compatibility with polymers, and reduces their photocatalytic activity. The resulting double-shell-structured NPs were then utilized as anti-UV additives to prepare PVC composite films through a casting process. The resulting composite film displayed significantly enhanced anti-UV efficiency and improved NP dispersibility [145]. Polyamide/modified-TiO_2_ (PA/m-TiO_2_) NCs were prepared by Dinari et al. (2017) [146]. Initially, they synthesized a soluble aromatic PA using a direct polycondensation reaction, yielding good results with moderate inherent viscosity. To improve the dispersion of TiO_2_ NPs and enhance their interactions with the polymeric matrix, the surface of TiO_2_ was modified using APTES. The functionalized NPs were then incorporated into the polymer matrix. Thermal stability of the NCs was found to increase with increasing the m-TiO_2_ NP content, as indicated by the TGA data shown in Figure 10a [146]. Silanes were used to link the MO NPs with rubber. Biuk Afshari et al. (2022) [147] developed a NC adhesive by incorporating cerium oxide (CeO_2_) NPs into nitrile rubber (NBR) to improve its mechanical strength and resistance to corrosion. To prevent the agglomeration of the NPs and promote their dispersion in the rubber matrix, they used TESP to graft CeO_2_ NPs at various concentrations. The hydrothermally steamed CeO_2_ NPs with the highest silane grafting ratio were found to be the most effective in enhancing the anticorrosion properties and cathodic dis-bonding of NBR-based adhesives. Additionally, the modified and steamed NPs facilitated the crosslinking of the NBR compound and improved the interfacial interactions between the rubber chains and the NP surface [147]. Jang et al. (2021) [148] utilized intense pulsed light (IPL) sintering to create a Si solar cell electrode using a paste made of oxidized Cu NPs. They improved the dispersion of the particles in the paste and their electrical conductivity by modifying the surface of the CuO NPs with a silane dispersant, specifically APTMS, as illustrated in Figure 10b. Their study revealed that the modified CuO NPs paste displayed excellent dispersion characteristics, enabling high reduction and sintering uniformity of multiple IPL-sintered Cu NP electrodes [148]. Jaramillo and colleagues (2019) [149] investigated the potential of using tannin as a corrosion inhibitor for steel ASTM A36. To achieve this, they incorporated tannin extract into an epoxy resin, along with ZnO NPs functionalized with APTES, at concentrations of 1%, 3%, and 5% *w*/*w*. The study findings revealed that the corrosion current density of the steel significantly decreased and increased its hydrophobicity. Moreover, electrochemical impedance spectroscopy (EIS) analysis demonstrated that the coatings with surface-modified NPs provided excellent protection against corrosion [149]. The impact of incorporating nanoscale magnesium oxide (MgO) into epoxy-based composites on their dielectric properties over a range of temperatures and frequencies was investigated by Hornak et al. (2018) [150]. They added GPTMS to MgO NPs in the case of an optimal filler loading, which established covalent interface links to prevent phase separation. The addition of the coupling agent led to improved dielectric properties of the entire composite and resulted in higher volume resistivity values, which could be attributed to a higher degree of filler dispersion in the matrix [150]. MPS was utilized by Aamer et al. (2021) [151] to enhance the dispersion of ZnO NPs within thin polyacrylonitrile fibers. A schematic diagram of the ZnO modification process with MPS is shown in Figure 10c. The nanofibers that were produced with silane-modified ZnO NPs exhibited significant enhancements in the specific surface area, surface roughness, and fiber porosity when compared to the nanofibers that were produced with unmodified ZnO NPs. Additionally, the modified ZnO/PAN nanofiber composite demonstrated exceptional antibacterial activity [151]. In another study Da Silva et al. (2019) [152] investigated how the size and surface of ZnO NPs (ZnO NP) affect their antibacterial activity against Staphylococcus aureus and Escherichia coli. They produced ZnO NPs using the sol–gel method, and then they modified the surface with GPTMS. This surface modification enabled the dispersion of the NPs in water, making them more useful. They found that the antibacterial activity increased as the particle size decreased [152]. In another study, vinyltrimethoxysilane (VTMO)-modified SiO_2_ NPs have been explored for enhancing two-component High Solid (2K HS) hybrid clearcoats (CC) for automotive applications, enabling low-temperature curing. These characterization techniques have been confirmed as delivering successful silane modification, while DMA has revealed that incorporating 1.75 phr VTMO-modified SiO_2_ improves clearcoat toughness by creating a low-density region within the polymer matrix. However, higher nanofiller content led to agglomeration and diminished performance. These findings highlight the potential of silane-modified NMs in optimizing clear coat formulations for improved mechanical properties and durability in the automotive industry [153]. Furthermore, silanized ZnO has been incorporated into benzoxazine-based composites for enhanced electromagnetic (EM) shielding. FTIR has been confirmed to deliver successful silane modification, while DSC and TGA have showed improved thermal stability. DMA has indicated an increased storage modulus and reduced glass transition temperature (T_g_). The composites have demonstrated excellent EM absorption, achieving reflection loss (RL) values up to −50 dB, thus highlighting the potential of silane-modified NMs for advanced shielding applications [154].

### 6.6. Other NMs

**Figure 11:**
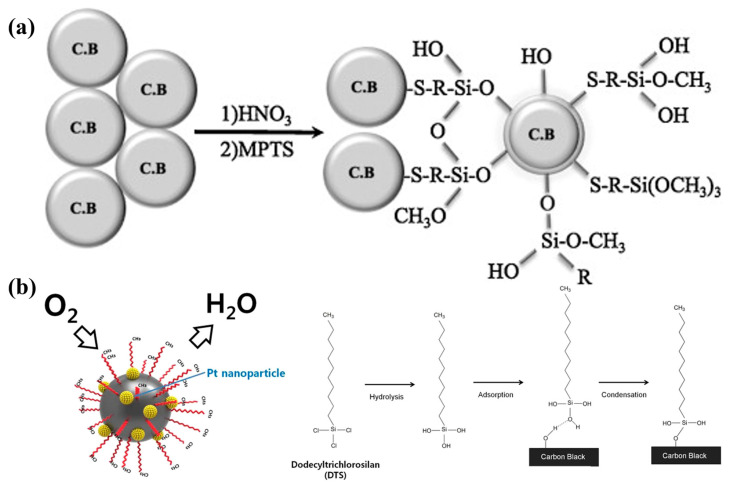
(**a**) CB surface modification by MPTS [157]. (**b**) Schematic diagrams of the step-by-step processes to create a DTS coating on carbon black and the preparation of Pt supported on DTS-coated CB [158]. All figures were reproduced with permission.

Silane modifications are also being carried out in other nano-materials, like metal NPs, CB, nanofibers, etc. Although there are not many studies being conducted with these materials, Xianxue et al. (2012) [155] conducted a modification of Ag NPs using a silane coupling agent, MPS. These modified Ag NPs were then employed as a conductive filler to prepare a conductive adhesive in epoxide resin vehicles at a temperature of 180 °C. They carried out modifications on Ag NPs to achieve uniformly dispersed Ag NPs. These modified NPs were later incorporated into epoxy resin, resulting in the preparation of a conductive Ag nano-paste. The Ag powders, after modification, exhibited excellent dispersal with an average size of around 20 nm. The MPS molecules have the capability to be adsorbed onto the surface of Ag particles. The bulk resistivity of the conductive adhesive measured 2.5 × 10^−3^ Ωcm. The adsorption of MPS molecules on the surface of Ag particles significantly enhances the conductivity of the conductive adhesives. When compared to the control sample, the modified Ag conductive adhesives demonstrated an impressive 3–5 times improvement in bulk conductivity [155]. The study conducted by Rehacek et al. (2019) [156] was centered on the deposition of Au NPs to enhance the properties of MO films (MOF) for hydrogen gas sensing. The researchers utilized MPS for silane functionalization on Au NPs to improve the properties of MOF because they can form a permanent linkage between Au NPs and underlying inorganic materials. The results showed that MOF with Au NPs exhibited more than twice the sensitivity for 100 ppm of H_2_ at 200 °C compared to that of the MOF [156]. Atif et al. (2013) [157] performed a thermal modification of CB by grafting silane moieties onto its surface. The purpose of this modification was to achieve molecular dispersion and prevent macroscopic phase separation in various solvents and monomers. CB is a fascinating material that captures the interest of both researchers and technologists due to its widespread application as a cost-effective industrial filler. This study aimed to functionalize the surface of CB using organo-alkoxysilanes, specifically the MPS shown in Figure 11a, to enhance compatibility, improve dispersion, and achieve better overall performance. As a result, the modified particles exhibited an increased negative surface charge and a larger surface area. The surface modification has a profound effect on dispersing the m-CB in any type of solvent, whereas particles treated solely by an oxidation process remain non-dispersible in a polar media [157]. Lee et al. (2015) [158] conducted functionalization of hydrophilic CBs through self-assembled coatings of dodecyltrichlorosilane (DTS). These modified CBs were utilized as catalyst supports for proton exchange membrane (PEM) fuel cells. The chlorosilane groups present in DTS underwent a reaction with the OH groups on the surface of CB, as shown in Figure 11b. This successful reaction led to the effective coating of DTS on CB, transforming the surface of CB from hydrophilic to hydrophobic. Homogeneously distributed platinum (Pt) NPs were dispersed on DTS-coated CB, and, importantly, the DTS coating did not adversely affect or poison the Pt catalyst. The incorporation of DTS-coated CB enhances the hydrophobicity of the catalyst layer, leading to improved mass transfer rates of reactant gas. This improvement results in better fuel cell performance. Corrosion tests have revealed that the resistance to electrochemical carbon corrosion is significantly higher for Pt/DTS-CB compared to Pt/CB. This improved resistance is attributed to the hydrophobic modification of CB achieved by DTS coating [158]. Table 2 summarizes the silane-modified NMs integrated with various materials.

**Table 2:** Summary of silane-modified NMs integrated with various materials.

S. No	Types of NM	Silane Modifier	Substrates Integrated with Silane Modified NM	References
1	Fullerene	APTES	TPU	[92]
		APTES	Montmorillonite	[94]
		APTES	Epoxy resin	[95,96]
2	CNT	GPTMS	Epoxy resin	[76]
		APTES	Cementitious matrix	[77]
		APTMS	Epoxy resin	[83]
		APTES	PP, PVC	[104]
		APTES	TPU	[10]
		VTES	LLDPE	[105]
		TESPT	NR matrix	[106]
		p-CMPTMS	Glass	[108]
		APTES	POM	[109]
		APTES	SiO_2_ NPs	[110]
		APTES	PA66	[111]
		AEAPTS	Epoxy resin	[112]
		HDTMS	Ethyl cellulose	[113]
		APTES	UD Kenaf	[114]
		BTESPA	Epoxy resin	[115]
3	Graphene	APTES	PVDF	[78]
		MPS	N-isopropylacrylamide	[123]
		APTES, GPTMS, MPS	PET	[124]
		APTMS	SR matrix	[126]
		APTES	Epoxy resin	[127]
4	GO	VTES	Vinyl-ester resin	[86]
		APTES	Cement	[159]
		APTES	PU	[131]
		APTES	Nylon 66	[132]
		AAPS	PA6	[133]
		ETEOS	PI	[134]
		APTES	PAN	[135]
		APTMS	Geopolymer	[136]
		APTES, TEOS	Epoxy resin	[137]
		TMSPED	Epoxy resin	[138]
		GPTMS	Epoxy resin	[85,139]
		TESPIC	PUU	[160]
		TESPIC	Cerium marix	[161]
		APTES	LDH	[162]
		AEAPTS	Glutaraldehyde	[163]
5	MO NP			
	ZnO	TEOS, APTES	PVC	[145]
	TiO_2_	APTES	PA	[146]
	CeO_2_	TESPT	NBR	[147]
	CuO	APTMS	Solar cell electrode	[148]
	ZnO	APTES	Epoxy resin	[149]
	MgO	GPTMS	Epoxy resin	[150]
	ZnO	MPS	PAN	[151]
	Al_2_O_3_	APTES	Epoxy resin	[84]
	SiO_2_	VTMO	Polyurethane	[153]
	ZnO	APTMS	Polybenzoxazine	[154]
6	Other NMs			
	Ag NPs	MPS	Epoxy resin	[155]
	Au NPs	MPS	MOF	[156]
	CB	MPS	Solvent	[157]
	CB	DTS	Pt NPs	[158]

## 7. Applications

Silane-modified NMs have become a diverse and dynamic class of compounds that have extensive uses in various sectors. By introducing silane groups onto the surface of NMs, enhanced dispersion, improved adhesion, and heightened compatibility with various matrices are achieved. This renders them as valuable additives with vast applications across a wide range of industries. They have applications in the textile industries, in water treatment, catalysis, biomedical application, coating agents, etc. Silane-modified NMs have sparked a revolution across diverse industries, presenting customized solutions to a wide array of engineering challenges. Some of the applications of silane-modified NMs are evaluated in this section.

### 7.1. Textile Industries

**Figure 12:**
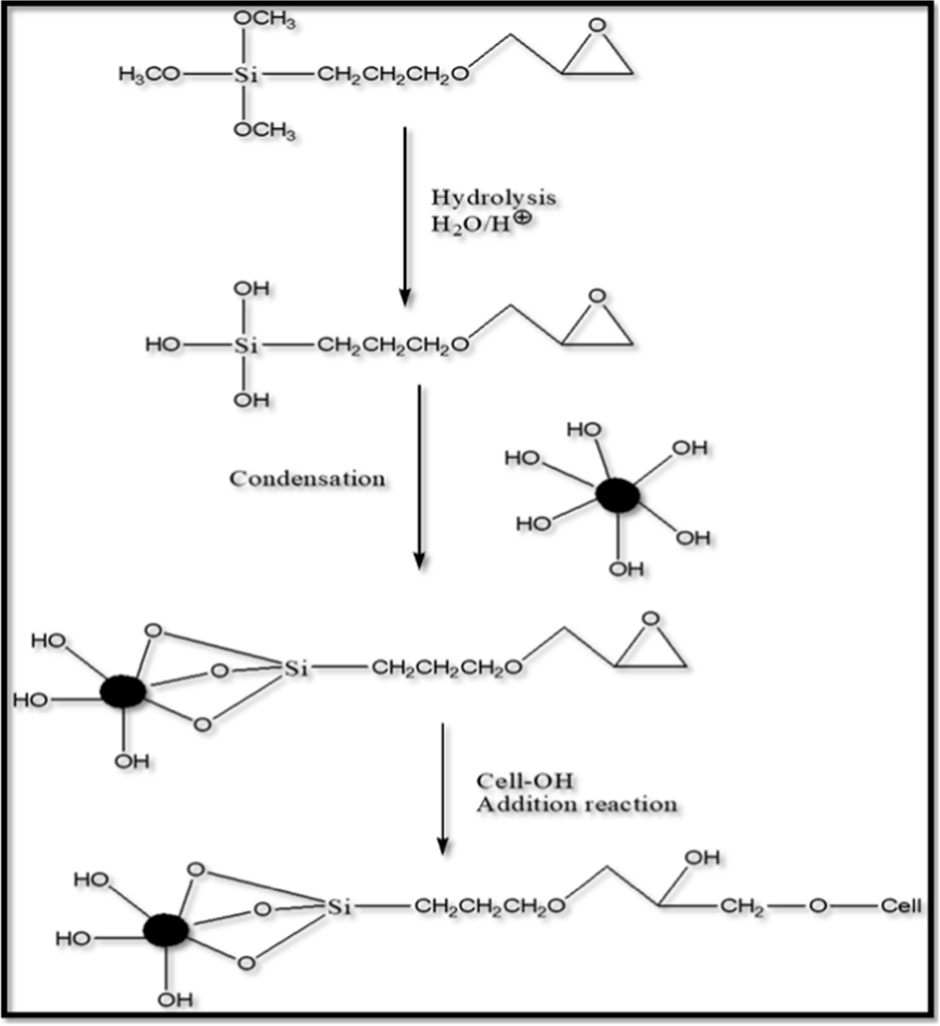
Diagram showing the molecular-level bonding of modified ZnO NPs onto cotton fabric [170]. The figure was reproduced with permission.

Textiles are among the most widely utilized materials across various industries and households. Recently, there has been a great deal of interest in the surface modification of textiles in order to give numerous functionalities, such as antibacterial [164], superhydrophobic [165], UV blocking [166], and flame-retardant [167] properties. Silane-modified NPs offer a wide range of uses across the textile industry because of their exceptional characteristics and potential advantages [168,169]. Afzal et al. (2022) [170] utilized GPTMS to modify ZnO NPs and developed an anti-bacterial cotton fabric using the pad dry cure method. The schematic illustration of the linking of ZnO NPs to cotton fabric is shown in Figure 12. The NPs were reacted with the silane portion of GPTMS under acidic conditions. The use of GPTMS increased the binding of NPs to the cotton fabric without adversely affecting the inherent properties of the fabric. The resulting fabric exhibited excellent antiviral and antibacterial properties, which were maintained even after undergoing severe industrial washing [170]. Agrawal et al. (2020) [168] demonstrated efficient manufacturing of a durable super-hydrophobic antibacterial fabric materials using an easy technique. Silane coupling agents were used as cross-linkers to attach copper oxide NPs (CuO NPs) onto the surface of cotton fabric coated with polydimethylsiloxane (PDMS) for increased durability. Aminoethyl aminopropyltrimethoxysilane (AEAPTMS), APTES, and MPS are three silane cross-linkers that have been studied. The material’s super-hydrophobicity and anti-bacterial activity were good as-prepared. When exposed to abrasion, ultrasonic cleaning, and severe chemical conditions, the materials demonstrated greater resilience. Even after ultrasonic cleaning, the material’s anti-bacterial properties remained intact. The textile’s elasticity and breathability were also kept intact [168].

### 7.2. Water Treatment

**Figure 13:**
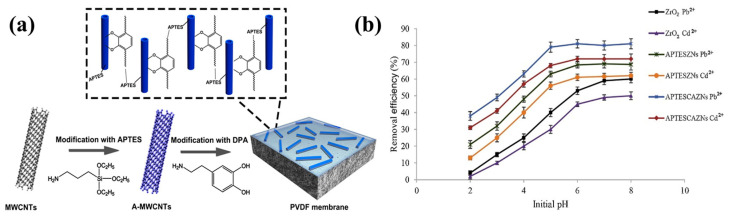
(**a**) Schematic illustration for the fabrication of dopamine/A-MWCNTs decorated with a superhydrophilic PVDF membrane [172]. (**b**) The effect of pH on the adsorption of metal ions by ZrO_2_ NPs, APTESZNs, and APTESCAZNs [175]. All figures were reproduced with permission.

Water contamination now mostly arises from a variety of chemical pollutants. The refinement processes are usually costly and hazardous. Adsorbent materials are effective in removing both organic and inorganic impurities, making them suitable options for refinement processes. The emergence of silane-modified NMs as adsorbents has helped water treatment. Silane-modified NMs can be used in heavy metal removal, organic pollutant removal, oil removal, etc. [171]. Yang et al. (2017) described a straightforward and adaptable approach to create a super-hydrophilic PVDF membrane using copolymerized dopamine, as shown in Figure 13a. In order to enhance the surface wetting properties of the PVDF membrane, MWCNTs have been integrated into the membrane due to the extraordinary properties of CNT. However, due to the tendency of MWCNTs to aggregate in polymer membranes, their dispersibility is poor; hence, they functionalized MWCNTs with APTES to enhance the interfacial interaction and compatibility between the NPs and the membranes. The resulting PVDF membranes exhibited excellent super-hydrophilicity and underwater superoleophobicity. As a result, these membranes exhibited significant potential for separating oil-in-water emulsions and water filtration applications [172]. For the purpose of removing arsenic ions from aqueous solutions, Tamaddoni et al. (2019) developed a new polyurethane foam (PU) NC adsorbent based on magnetic iron-oxide NPs that have been modified with silane (Fe_3_O_4_@APTES). A significant adsorption performance (q_max_) and a 95% removal efficiency were displayed by the modified magnetic PU foam throughout the course of a 4 h contact period. Higher arsenic removal capabilities from drinking water are offered by modified iron oxide’s capacity to interact with arsenic through both an ion exchange method and adsorption. Additionally, through employing an external magnet field, the newly developed NC foam can be readily removed from an aqueous solution [173].

Through using magnetic NPs with a silica coating and an amine functional silane APTES (ASMNPs), Naeimi et al. (2017) created a magnetically recoverable adsorbent. The magnetic NPs with amino modifications that had been created were employed as an absorbent to remove diazinon from water. The batch approach was used to determine adsorption. In the presence of 2.5 mg of the ASMNPs, 84% of diazinon, in 5 mg/L of initial diazinon concentration, was eliminated within half an hour [174]. Majedi et al. showed the fabrication of a new adsorbent for the removal of Pb (II) and Cd (II) as heavy metal ion models from water using (APTES)/citric acid (CA) modified zirconia NPs (APTESCAZNs). Studies were conducted into how the pH in the solution influenced the effectiveness of removing Pb^+2^ and Cd^+2^. It was explained that aqueous solutions should have a pH of 6 in order to effectively remove Pb^+2^ and Cd^+2^. The greatest observed adsorption capacities for Pb^+2^ and Cd^+2^ were 172.4 and 90.9 mg/g, respectively. Additionally, in Figure 13b, the removal efficiency of Pb^2+^ and Cd^2+^ ions at various pH by both primary and modified zirconia NPs is depicted. It was noted that, as the initial pH of the solution increased from 2 to 6, the removal efficiency of all synthesized particles rose. However, beyond a pH of 6, the adsorption percentage remained constant [175].

### 7.3. Painting and Coatings

**Figure 14:**
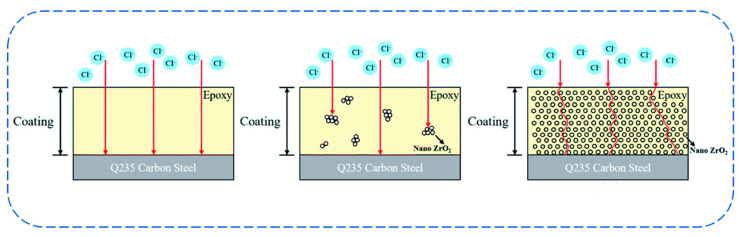
The corrosion protection mechanisms of three types of coatings: pure epoxy, 3–4 wt.% zirconia, and 1–2 wt.% zirconia [183]. The figure was reproduced with permission.

Silane-modified NMs exhibit distinct properties and functionalities, making them highly sought after in the realm of paintings and coatings, where they have been widely utilized for diverse applications [176,177]. By integrating these NMs into paints and coatings, their overall performance is substantially enhanced, leading to a wide range of advantages and benefits, such as improved adhesion [178], enhanced durability, super-hydrophobicity [179], corrosion resistance [180], and anti-microbial properties [181]. Wang et al. (2019) presented a new approach for enhancing the efficiency of natural fiber-based composites by coating CNT on the ramie fibers. To avoid the agglomeration of CNTs in suspension and on coated fiber surfaces, a surface functionalization of CNTs was carried out using a silane coupling agent, APTES. The resultant silane-coated CNTs were covalently bonded to the fiber surfaces rather than through a weak van der Waals force [182]. Shu and colleagues (2019) utilized GO as a framework, which was subsequently modified using a silane coupling agent, APTES (KH550), to produce modified GO. By means of chemical grafting, composite coatings of GO and waterborne polyurethane, employing varying proportions, were prepared. Structural characterization tests were conducted to evaluate these coatings. The findings indicate that the silane coupling agent effectively modified the GO, leading to an increased presence of oxygen-containing functional groups on the surface of the modified GO. Incorporating the modified GO into the coatings significantly improved their corrosion resistance. Optimal corrosion resistance was achieved with a mass fraction of 0.03%, as evidenced by only 5% corrosion occurring after 48 h, while the total corrosion time extended to 120 h [180]. In Wu et al.’s (2022) study, an epoxy coating containing ZrO_2_ NPs was prepared and applied onto the surface of Q235 mild steel through electrostatic spraying. The ZrO_2_ NPs were modified with APTES to ensure their even distribution in the epoxy coating. As shown in Figure 14, pure epoxy is not capable of blocking the corrosion medium that directly penetrates into the metal, and it results in corrosion. When zirconia NPs are loaded in large amounts, agglomeration happens and corrosion resistance will be poor. When the composite coating is low, there will be an even distribution of NP, which increases the time for the corrosion medium to reach the metal surface. This leads to enhanced corrosion resistance. Their findings suggest that incorporating a suitable quantity of APTES-modified ZrO_2_ NPs into an epoxy coating significantly enhances the corrosion resistance of the Q235 surface, and it also improves its protective performance [183]. Javadi et al. (2019) examined how the use of silane-treated nano ZnO (nZnO) affects properties and interfacial interactions in acrylic/carbon steel systems. They synthesized nZnO particles, which were then treated with 3(2-amino ethyl amino) propyl trimethoxysilane and dispersed in acrylic resin to create a NC coating applied to a carbon steel substrate. They found that the inclusion of silane-modified nZnO in the acrylic coatings up to 1% wt. led to an increase in protective performance [184]. Li et al. (2021) developed a simple method to create a superhydrophobic coating based on acrylic resin. The approach involved modifying ZnO NPs with HDTMS to obtain hydrophobic ZnO, which was then uniformly mixed with acrylic resin. The resulting mixture was applied onto an aluminum sheet and cured to form a coating. Optimal hydrophobicity was achieved when the modification agents were used at a concentration of 10 wt.% [179]. Chen et al. (2019) developed a new composite material called polyvinyl sesquisiloxane PVSQ-GO by synthesizing silane-functionalized GO through a hydrolysis condensation reaction of VTES monomers on the surface of GO. The resulting PVSQ-GO composite demonstrated excellent dispersion in an environmentally friendly waterborne polyurethane coating. Electrochemical impedance spectroscopy analysis revealed a significant enhancement in the anti-corrosion properties of the waterborne polyurethane coating when embedded with 0.5 wt.% of the PVSQ-GO composite [185].

### 7.4. Catalysis

**Figure 15:**
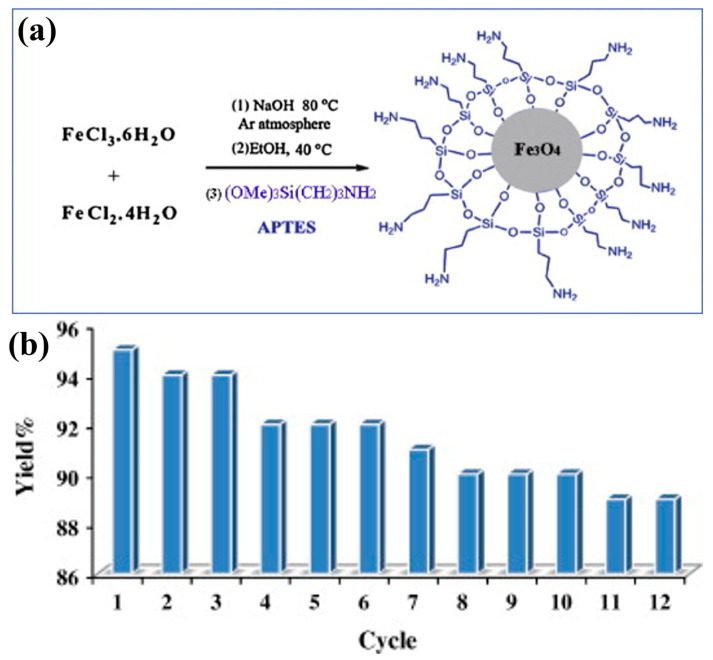
(**a**) Synthesis of Fe_3_O_4_-MNPs and their surface modification by APTES. (**b**) Reusability and recycling of nano-catalyst for preparation of 2-amino-4H-chromene components [187]. All figures were reproduced with permission.

Silane-modified NMs have demonstrated excellent potential for catalysis. Silane compounds are used to functionalize NMs, which improves their catalytic abilities and creates new possibilities for a variety of catalytic applications. They have been used in heterogeneous catalysis, photocatalysis, electrocatalysis, etc. [186]. Chen and colleagues (2022) developed a novel catalyst consisting of Pt-Ni NPs supported on GO modified with a silane-based substance called APTES. This modification process resulted in the formation of amine-functionalized GO (NH_2_-rGO), which enhanced the interaction between the carbon support and the metal catalyst. The resulting catalyst, referred to as PtNi/NH_2_-rGO, exhibited remarkable catalytic activity and improved stability compared to the catalyst without APTES modification [186]. Safari et al. used an amino-silane-modified Fe_3_O_4_ NP (MNPs-NH_2_) heterogeneous nano-catalyst in a one pot in a three-component coupling process of aldehyde, malononitrile, and resorcinol to successfully synthesize 2-amino-4H-chromenes. The magnetic NPs were modified using APTES, as shown in Figure 15a. This approach has a number of benefits, including a small reaction time, an easy set-up, great yields, ease of separation, recyclability of the magnetic nano-catalyst, and the capacity to withstand a wide range of reagent substitutes. It has been observed that the nano-catalyst can be recycled after many catalytic cycles (Figure 15b) [187]. Azizi et al. produced magnetic NPs with thiamine (VB1), which was modified using GPTMS moiety, grafted onto them (Fe_3_O_4_@SiO_2_@GMSI-VB1), and then used them as a magnetically recoverable catalyst for an effective and environmentally friendly production of 2,3-dihydroquinazolin-4(1H)-ones under an aqueous environment. Fe_3_O_4_@SiO_2_@GMSI-VB1 gives a unique, stable, and long-term approach to the collection of 3,4-dihydroquinazolin-2(1H)-one derivatives by exhibiting enhanced catalytic activity over the condensation process of the 2-aminobenzamide with an aldehyde/ketone under an aqueous medium. With a wide range of substrates and comparable conditions, several desired products have been produced with high-to-outstanding yields (72–95%) and selectivity [188].

### 7.5. Biomedical Applications

**Figure 16:**
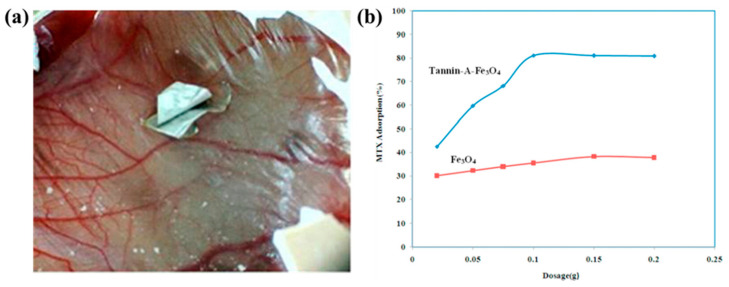
(**a**) Chick chorionic allantoic membranes (CAM) assay of HA/CNT, showing the vasculature response of the prepared materials [192]. (**b**) The influence of varying dosages of Fe_3_O_4_ and Tan-A-Fe_3_O_4_ on MTX adsorption [193]. All figures were reproduced with permission.

Due to their adaptable surface chemistry and biocompatibility, silane-modified NMs have demonstrated immense possibilities in several biological applications [189,190]. Silane-functionalized NMs enable precise targeting, regulated drug delivery, and improved interactions to biological systems. Levenez et al. (2021) conducted a study where they enhanced the interaction between GO nanosheets and the poly(methyl methacrylate) (PMMA) matrix by treating GO with MPS. The modified GO was then incorporated into acrylic bone cement formulations at varying percentages (0.1%, 0.5%, and 0.75%). They observed notable improvements in the mechanical properties of the resulting composites. Interestingly, their findings indicate that, when a 1 wt.% concentration of the silane coupling agent was utilized during the surface treatment of GO, the material exhibited the most favorable mechanical performance. In terms of biological characteristics, tests for osteoblast cell viability and hemocompatibility were conducted. The findings showed that either the presence of GO or that of silane compromises the cytocompatibility and hemocompatibility of formulations for acrylic bone cements [191]. Khan et al. (2017) created bioactive electrospun fibers by in situ precipitation of nano-hydroxyapatite (nHA) along with different concentrations of silane-functionalized MWCNTs. They used MPS for silanization and mixed with polyvinyl alcohol (PVA) before being electrospun to form the fibers. To evaluate the biocompatibility of the fibers, the researchers conducted a chick chorioallantoic membrane (CAM) assay and showed that the HA/CNT electrospun fibers exhibited excellent biocompatibility; thus, it was appropriate for clinical application (Figure 16a) [192]. A new drug carrier promoting the adsorption of methotrexate (MTX) was created by Langeroudi et al. (2018) using magnetic NPs modified with tannins and loaded with APTES (Tan-A-Fe_3_O_4_). Tan-A-Fe_3_O_4_ had a maximum MTX adsorption percentage of 91%, as shown in Figure 16b, and a maximum monolayer adsorption capacity of 55.55 mg/L. Tan-A-Fe_3_O_4_ nano-adsorbent is a feasible and environmentally friendly carrier for the successful removal of MTX from an aqueous solution [193]. APTES-modified Fe_3_O_4_ NPs have been used by Zhang et al. (2018) as drug carriers in the administration of cytosine-phosphate-guanine (CpG) as a therapeutic strategy for the treatment of cancer. The outcomes show that APTES-modified Fe_3_O_4_ NPs have high stability, low toxicity, and elevated CpG carrying capacities in both vitro and in vivo [194].

### 7.6. Membrane Applications

**Figure 17:**
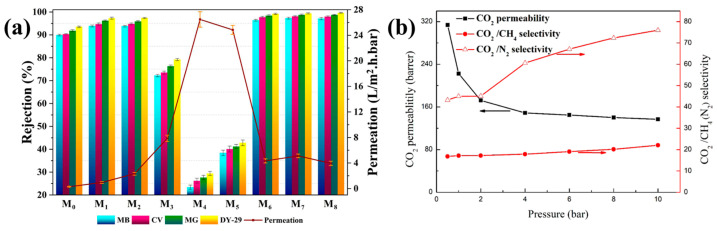
(**a**) Dye removal performance of M7 membrane [196]. (**b**) The impact of different feed gas pressures on the gas separation performances of the Pebax/f-GO-0.7% membrane [197]. All figures were reproduced with permission.

Silane-modified NMs also display use in other applications, such as in the field of diffusion membrane systems [195]. Mahdavi and colleagues (2023) conducted a study in which they developed nanofiltration (NF) membranes by integrating different proportions of polyether sulfone, TPU, and GO nanosheets functionalized with APTES. The researchers observed that incorporating TPU and embedding GO-APTES within the PES matrix led to enhancements in the tensile strength and elongation at the break of the fabricated membranes. Additionally, it was determined that the membrane containing 2% wt TPU and 0.3% wt GO-APTS (M7 membrane) exhibited superior dye removal performance of common dyes, such as MB, CV, MG, and DY-29 (Figure 17a) [196]. In this study, Zhang et al. (2019) successfully developed mixed matrix membranes using amino-silane as both an interface moderator and a CO_2_ carrier. They achieved this by incorporating APTS functionalized GO nanosheets into a Pebax matrix. Compared to pure GO, the amino-silane functionalized f-GO nanosheets demonstrated improved interfacial compatibility with Pebax, resulting in a homogeneous dispersion of fillers within the polymer matrix. Furthermore, the Pebax chains penetrated the Si-O-Si network, significantly enhancing the mechanical strength of the membranes. The f-GO effectively disrupted the arrangement of the original polymer segments, thereby reducing the crystallinity of Pebax and creating additional CO_2_ transport pathways. Additionally, the introduction of CO_2_-philic amino groups greatly improved the solubility and selectivity of CO_2_. These membranes exhibited exceptional performance under humid conditions, with a CO_2_ permeability of 913.0 Barrer, a CO_2_/CH_4_ selectivity of 40.9, and a CO_2_/N_2_ selectivity of 71.1, as shown in Figure 17b [197]. The study conducted by Wasim et al. (2021) aimed to investigate the impact of functionalized graphene, when synthesized via a modified Stöber method using VTES, on the construction of NF membranes for the removal of azo dyes. Different concentrations of VTES-G were introduced to a crosslinked polymeric solution to enhance dye rejection. The results showed that the addition of VTES-G led to significant reductions in dye concentrations, with rejection rates of 97.8% for Congo Red, 99.9% for Reactive Black 5, and 96.8% for Reactive Orange 16. Their study also revealed that the modification process improved the antifouling properties of the membranes, resulting in a decrease in the microbe adsorption on the membrane surfaces [198]. Zhang et al., in 2023, prepared a super-hydrophobic microporous layer (MPL) using a functionalized CB. The CB was modified using MPS, which helps in achieving enhanced hydrophobicity for the MPL. The MPL developed using the modified CB demonstrated improved water, as well as gas, permeability. This MPL is capable of rendering a proton exchange membrane fuel cell (PEMFC) that has more of an outstanding electrochemical performance than the conventional method [199].

### 7.7. Energy Harvesting Application

Energy harvesting technologies, which convert ambient mechanical, thermal, or solar energy into usable electricity, are critical for powering next-generation electronics sustainably [200]. Silane-modified NMs enhance energy harvesting properties by improving interfacial interactions, charge transfer, and stability in energy harvesting devices, such as piezoelectric, triboelectric, and photovoltaic devices. Silane coupling agents improve the dispersion of NMs in polymer matrices or solvents. Better dispersion ensures uniform energy harvesting by reducing agglomeration [9]. In photovoltaics, this uniform dispersion enhances light absorption and charge generation, while, in piezoelectric composites, it improves stress transfer [201,202]. Silanes also help in facilitating electron transfer between NMs and electrodes in solar cells [203,204]. For triboelectric devices, silane-modified surfaces enhance charge [205]. This section has reviewed various energy harvesting applications of silane-modified NMs.

#### 7.7.1. Triboelectric Nanogenerator (TENG) Application

**Figure 18:**
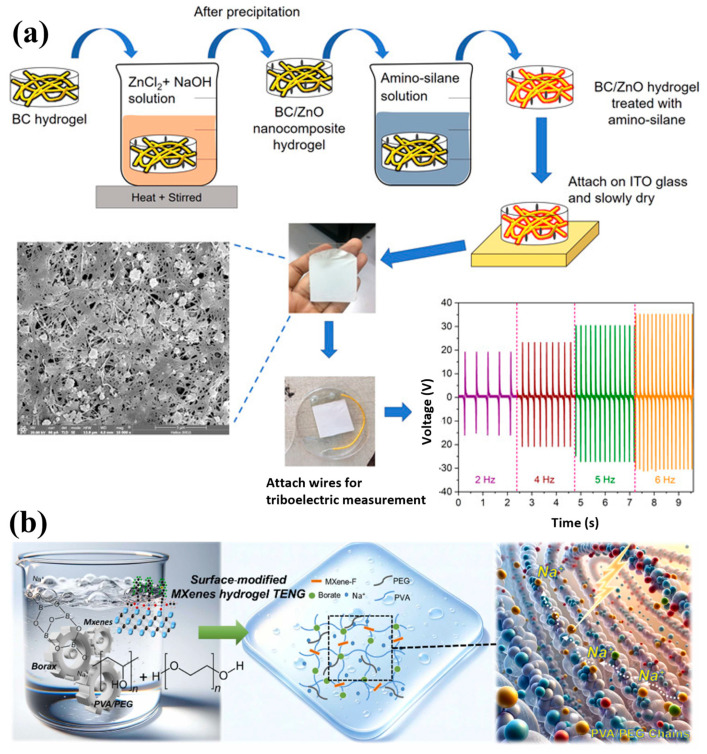
(**a**) Fabrication process of the BC/ZnO (ZBC) NC hydrogel when it is treated with amino-silane, as well as its adhesion on ITO glass for TENG measurement [210]. (**b**) The schematic diagram illustrates the process of the surface functionalization of MXene nanosheets within a borax-PVA/PEG hydrogel used in TENGs [205]. All figures were reproduced with permission.

Mechanical energy is one of the most abundant and reliable energy sources in our environment, offering stability, accessibility, and long-term availability [206]. In 2006, Wang et al. introduced the first triboelectric nanogenerator, providing an innovative solution for efficiently converting mechanical energy into usable electrical power [207,208]. Silane-modified NMs enhance the triboelectric performance of TENGs by improving surface charge transfer, adhesion, and dielectric properties [209]. Bacterial cellulose (BC)-based bio-triboelectric nanogenerators (bio-TENGs) offer promising potential for self-powered electronics, but conventional processing methods often degrade their nanostructure and crystallinity. To address this, Jakmuangpak et al., in 2020, prepared silane-modified ZnO-impregnated BC (ZBC) NCs, enabling direct adhesion to indium tin oxide (ITO) substrates without adhesives. This approach preserves BC’s nanostructure while enhancing surface roughness and polarizability, leading to improved triboelectric performance. The optimized bio-TENG achieved a Voc of 57.6 V, an Isc of 5.78 μA, and a power density of 42 mW/m^2^, demonstrating the effectiveness of silane modification in enhancing TENG output, as shown in Figure 18a [210]. To enhance the performance of hydrogel-based TENGs, silane-modified MXene nanosheets were incorporated into a polyvinyl alcohol (PVA)-polyethylene glycol (PEG) hydrogel matrix by Lai et al. in 2024 [205]. In addition, 3-chloropropyltrimethoxysilane (CPTMS) and perfluorooctyltriethoxysilane (FOTS) were used to modify MXene, forming Cl-MXene and F-MXene, which significantly improved ionic conductivity and the streaming vibration potential (SVP) effect. The Cl-MPPh and F-MPPh TENGs achieved a Voc of 190 V and 212 V, respectively, with enhanced charge density and electrostatic interactions. The F-MPPh TENG demonstrated high sensitivity to mechanical stimuli, making it promising for motion sensing applications. The schematic diagram illustrates the process of the surface functionalization of MXene nanosheets, as shown in Figure 18b [205]. In another study, fluoroalkylsilane (FAS) modification was utilized to enhance the energy harvesting performance of textile-based TENGs while maintaining thermal insulation and Joule heating properties. A multilayer-coated nylon fabric with Ag nanowires encapsulated in polydimethylsiloxane effectively converts human motion into electricity, achieving a peak power density of 2.8 W/m^2^. The FAS-modified film also improves thermal insulation by 8% and enables rapid Joule heating, demonstrating its potential for self-powered wearable applications [211]. Silane modification enhances the performance of cellulose-based TENGs by improving surface polarity and hydrophobicity. Triethoxy-1H,1H,2H,2H-tridecafluoro-n-octylsilane (PFOTES) was grafted onto cellulose nanofibrils (CNFs), significantly increasing triboelectric charge density and humidity resistance. PFOTES-CNF TENG retained a 70% output at 70% humidity and achieved double the short-circuit current compared to unmodified CNFs, demonstrating the effectiveness of silane functionalization in developing durable, high-performance bio-TENGs [212]. Khan et al., in 2024, developed a silane-modified Linde type A (LTA) zeolite integrated with PDMS to create a highly efficient and durable TENG for energy harvesting in harsh environments. The silane-coupled LTA/PDMS-based TENG demonstrated a high-output power density of 42.6 µW/cm^2^, with an Voc of 120 V and a Isc of 15 µA at 14 Hz. The device exhibited exceptional stability, maintaining performance over 30,000 cycles, making it suitable for long-term applications [209]. In a study conducted by Qu et al., in 2024, a superhydrophobic and flexible nanofiber membrane (HPP-NF) was developed for TENGs using electrospinning and spray-coating techniques. The membrane was fabricated from biodegradable polycaprolactone (PCL) doped with conductive NPs and modified with long-chain silane and SiO_2_ to enhance hydrophobicity and flexibility. The resulting HPP-NF exhibited excellent liquid repellency, self-cleaning properties, and stable electrical output in high-humidity environments, with an Voc of 150 V and a Isc of 11 µA [213].

#### 7.7.2. Piezoelectric Nanogenerator (PENG) Application

**Figure 19:**
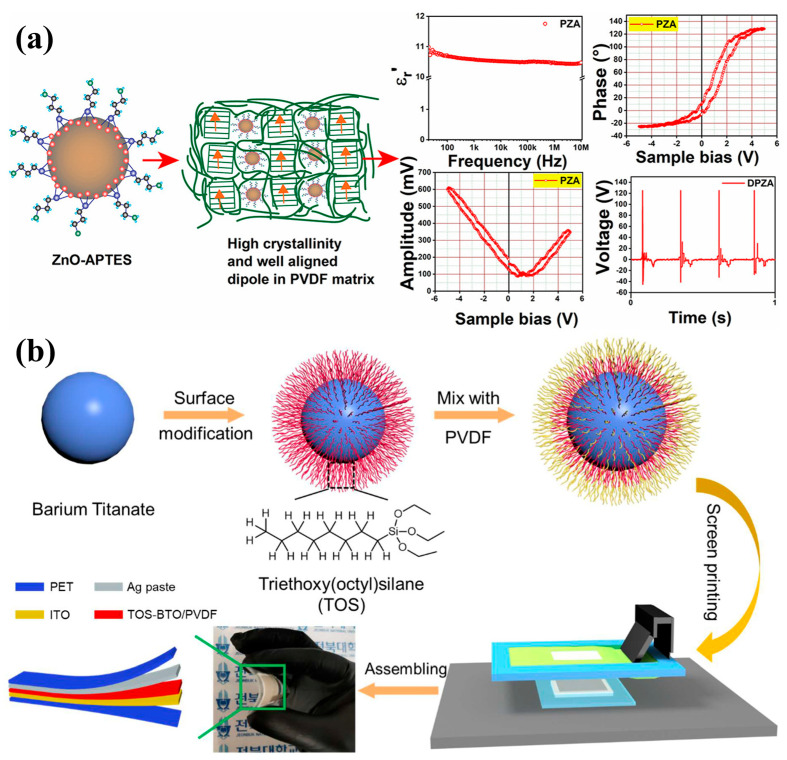
(**a**) Effect of APTES-functionalized ZnO NPs on β-phase crystallization, dipole moments, and crystallinity during low-temperature phase inversion compared to samples without and with ZnO–APTES NPs and their piezoelectric output performance [217]. (**b**) Schematic illustration of the fabrication process for the screen-printed TOS-BTO/PVDF piezoelectric sensor [221]. All figures were reproduced with permission.

Piezoelectric energy harvesting has emerged as a promising technology to convert ambient mechanical energy (e.g., vibrations and human motion) into electricity for powering microelectronics and IoT devices [214]. In 2006, Wang et al. pioneered the development of PENG based on ZnO nanowires, demonstrating their potential for scavenging energy from ambient mechanical sources [215]. Silane-modified NMs significantly enhance piezoelectric performance by improving the polarization alignment, stress transfer, and interfacial bonding within polymer matrices [216]. These advancements have enabled the development of high-efficiency, durable piezoelectric energy harvesters capable of powering small-scale electronics in diverse environments. Chandran et al., in 2021, significantly enhanced the piezoelectric performance of PVDF nanocomposites through the APTES functionalization of ZnO NPs combined with low-temperature phase-inversion processing, as shown in Figure 19a. The silane-modified ZnO NPs promoted the formation of PVDF’s electroactive β-phase, substantially improving dielectric, ferroelectric, and piezoelectric properties. The optimized PVDF/ZnO-APTES nanocomposite demonstrated exceptional energy harvesting capability, generating 125 V output voltage and 83 mW/cm^3^ power density—which is sufficient to power 50 LEDs simultaneously [217]. Bairagi et al., in 2019, developed a lead-free PENG using silane-modified potassium sodium niobate (KNN) nanorods incorporated into PVDF electrospun nanofibers. The silane modification prevented NP agglomeration, ensuring uniform dispersion and stronger interaction with the PVDF matrix, thereby significantly enhancing piezoelectric performance. The optimized PVDF/3% SM-KNN nanocomposite generated a high output voltage of ~21 V, a current of ~22 μA, and a power density of 115.5 μW/cm^2^, making it capable of powering LEDs and small electronics [218]. In a complementary study, Bairagi et al., in 2020, further investigated the role of surface modifications in optimizing PVDF’s electroactive properties by comparing three modifiers: APTMS, polyaniline (PANI), and PVP. The silane-modified KNN/PVDF composite exhibited superior performance, achieving a 98% β-phase content, a dielectric constant of 68, and a remarkable remnant polarization of 0.49 μC/cm^2^, thus far exceeding pure PVDF (0.001 μC/cm^2^) and untreated KNN composites (0.002 μC/cm^2^) [219]. In another study, Li et al., in 2022, developed a high-performance, fully 3D-printed PENG using triethoxyvinylsilane (TEVS)-modified barium titanate (BTO) NPs embedded in a PVDF-TrFE polymer matrix. The silane coating ensured a uniform dispersion of BTO NPs and strong interfacial bonding, significantly enhancing piezoelectric performance. The optimized PENG achieved an impressive output voltage of 54 V and a power density of 28.5 μW/cm^2^, maintaining stability over 13,500 cycles. Demonstrating practical applicability, the flexible device effectively harvested energy from human motion and enabled multipoint pulse detection [220]. Expanding beyond 3D printing, the same research group later demonstrated the versatility of silane-modified BTO composites by adopting a screen-printing approach for large-scale fabrication. In this study, Li et al. (2022) developed a high-performance screen-printed PENG using triethoxy(octyl)silane (TOS)-modified BTO NPs in a PVDF matrix, as given in Figure 19b. The TOS coating prevented NP agglomeration while enhancing interfacial bonding, leading to significantly improved charge transfer efficiency. The optimized device generated a 20 V output voltage and 15.6 μW/cm^2^ power density: representing 200% and 150% improvements over unmodified BTO/PVDF composites, respectively, while maintaining reliable performance over 7500 cycles. The screen-printed PENG effectively harvested biomechanical energy, showcasing excellent potential for wearable electronics applications [221].

#### 7.7.3. Photovoltaic Application

**Figure 20:**
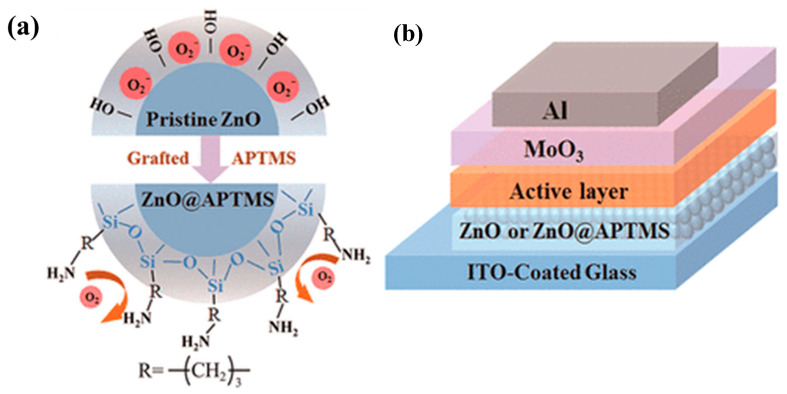
(**a**) Illustration displaying the schematic diagram of the ZnO and ZnO@APTMS NPs. (**b**) Diagram depicting the device architecture of the inverted solar cells [226]. All figures were reproduced with permission.

Solar energy stands as the most abundant renewable energy source available, providing clean and sustainable power with virtually unlimited potential [222]. The photovoltaic effect, first observed by Becquerel in 1839, enables direct conversion of sunlight into electricity through semiconductor materials [223]. Silane-modified NMs have demonstrated remarkable potential in several photovoltaic applications. Prabakaran et al., in 2015, significantly enhanced the performance of dye-sensitized solar cells (DSSCs) by developing a silane-modified TiO_2_/PEO/PVDF-HFP nanocomposite electrolyte. Through APTMS surface modification of TiO_2_ NPs, they achieved an order-of-magnitude improvement in ionic conductivity (7.02 × 10^−4^ S/cm vs. 7.94 × 10^−5^ S/cm for unmodified TiO_2_) by reducing polymer crystallinity and improving NP dispersion. When applied in a solid-state DSSCs with a natural gross dye sensitizer, the optimized electrolyte boosted key photovoltaic parameters—increasing open-circuit voltage by 52% (from 0.31 V to 0.47 V) and achieving an excellent 64% fill factor [224]. Building on these promising results, the researchers further optimized the system by fine tuning the TiO_2_ NP loading and investigating the electrolyte’s impact on the complete photovoltaic device performance. In their subsequent work, Prabakaran et al. (2015) developed a high-performance polymer blend electrolyte by incorporating 7 wt.% APTMS-modified TiO_2_ NPs into a PEO/PVDF-HFP matrix. This optimized composition achieved an ionic conductivity of 7.21 × 10^−4^ S cm^−1^, nearly 9 times higher than unmodified TiO_2_ composites, while simultaneously improving thermal stability and mechanical properties. The enhanced electrolyte enabled DSSCs to reach a 0.71 V *V*_oc_ and 2.84% power conversion efficiency, demonstrating how silane-functionalized NMs can dramatically improve photovoltaic performance through optimized polymer–electrolyte interfaces and reduced charge transport resistance [225]. Similarly, Sasi et al. developed highly efficient, flexible DSSCs using silane-modified TiO_2_ (M-TiO_2_) NPs that eliminated the need for binders or high-temperature sintering. The surface modification enhanced photocatalytic activity, achieving >95% methylene blue degradation within 10 min, while also improving photovoltaic performance, yielding a 4.1% solar conversion efficiency in DSSCs. Characterization revealed superior charge separation and stronger dye–TiO_2_ interactions in M-TiO_2_ compared to unmodified TiO_2_ [204]. In another study, Wei et al. (2018) created a buffer layer using APTMS-capped ZnO NPs (ZnO@APTMS). An illustration displaying the schematic diagram of the ZnO and ZnO@APTMS NPs is shown in Figure 20a. The incorporation of APTMS prevented the aggregation of ZnO NPs, resulting in excellent dispersibility in ethanol and long-term stability. These modified ZnO NPs promise high device performance, making them a desirable option for R2R-printed organic photovoltaics. Figure 20b, illustrated below, is the schematic representation of the device architecture for the inverted solar cells [226]. Table 3 shows some of the applications of silane-modified NMs.

**Table 3:** Summary of the studies on silane-modified NMs with different applications.

S. No	Type of NM	Silane Modifier	Application	References
1	ZnO NP	GPTMS	Textile industry	[170]
2	CuO NP	AEAPTMS, APTES, MPS	Textile industry	[168]
3	CNT	APTES	Water treatment	[172]
4	Fe_3_O_4_	APTES	Water treatment	[174]
5	ZrO_2_	APTES	Water treatment	[175]
6	Fe_3_O_4_	APTES	Water treatment	[173]
7	CNT	APTES	Paintings and coatings	[182]
8	GO	APTES	Paintings and coatings	[180]
9	ZrO_2_	APTES	Paintings and coatings	[183]
10	ZnO	AEAPTMS	Paintings and coatings	[184]
11	ZnO	HDTMS	Paintings and coatings	[179]
12	GO	VTES	Paintings and coatings	[185]
13	GO	APTES	Catalysis	[186]
14	Fe_3_O_4_	APTES	Catalysis	[187]
15	Fe_3_O_4_	GPTMS	Catalysis	[188]
16	GO	MPS	Biomedical application	[191]
17	CNT	MPS	Biomedical application	[192]
18	Fe_3_O_4_	APTES	Biomedical application	[193]
19	Fe_3_O_4_	APTES	Biomedical application	[194]
20	GO	APTES	Nanofiltration membrane	[196]
21	GO	APTES	Mixed matrix membranes	[197]
22	Graphene	VTES	Nanofiltration membranes	[198]
23	CB	MPS	Microporous layer	[199]
24	ZnO	APTES	TENG	[210]
25	MXene nanosheets	CPTMS	TENG	[205]
26	Ag nanowires	FAS	TENG	[211]
27	Cellulose nanofibrils	PFOTES	TENG	[212]
28	LTA zeolite	GPTMS	TENG	[209]
29	SiO_2_	HDTMS	TENG	[213]
30	ZnO	APTES	PENG	[217]
31	KNN nanorod	APTMS	PENG	[218]
32	KNN nanorod	APTMS	PENG	[219]
33	BTO	TEVS	PENG	[220]
34	BTO	TOS	PENG	[221]
35	TiO_2_	APTMS	Photovoltaics	[224]
36	TiO_2_	APTMS	Photovoltaics	[225]
37	TiO_2_	APTMS	Photovoltaics	[204]
38	ZnO	APTMS	Photovoltaics	[226]

## 8. Future Perspectives

Understanding the various attributes of surface modification of NMs provides a strong foundation for the development of advanced materials tailored for future technological applications. The advancement of eco-friendly and sustainable silane modification strategies is crucial, especially to address growing biomedical and environmental safety concerns. Future studies should focus on exploring novel NMs and assessing their compatibility with a wide range of silane coupling agents, which can lead to highly customized NC designs for specific functional needs.

Furthermore, the integration of artificial intelligence and machine learning approaches holds significant promise in accelerating the discovery and optimization of silane modifications, enabling data-driven predictions for targeted applications. Evaluating the long-term stability, durability, and recyclability of silane-modified NCs will also be key in enhancing their commercial viability and encouraging widespread industrial adoption.

Additionally, the potential of these materials in next-generation energy harvesting systems remains largely unexplored and presents a promising avenue for future research. The authors are encouraged to delve deeper into how silane-modified NCs can be engineered for integration into flexible and stretchable electronics, self-powered wearable devices, and autonomous environmental sensors. These materials, with their tunable surface properties and improved interfacial compatibility, could significantly enhance the performance and durability of PENG, TENG, and hybrid nanogenerators.

In particular, leveraging their mechanical flexibility, environmental stability, and chemical functionality could enable efficient energy harvesting from ambient mechanical sources, such as human motion, vibrations, wind, and flowing water. Such applications are crucial for powering low-energy electronics in remote or off-grid settings. Exploring these directions could bridge the gap between laboratory-scale material development and real-world deployment in Internet of Things (IoT) systems, wearable health monitors, and green infrastructure. Including such forward-looking insights would not only broaden the technological scope of the current work but also strengthen its relevance to the rapidly evolving field of sustainable and decentralized energy technologies.

## 9. Conclusions

In conclusion, this review paper sheds light on the significant progress and potential of silane modification of NMs and its broad spectrum of applications. The comprehensive analysis of silane chemistry and its interaction with diverse NMs has underscored its efficacy in tailoring surface properties for enhanced performance in NCs. The investigation of key parameters influencing silane modification has provided valuable insights into optimizing the process and achieving desired NC characteristics. The successful integration of silane-modified NMs in various industries, including textile industries, catalysis, water treatment, coatings, and biomedical fields, showcases their versatility and promising impact on advanced material development. Furthermore, their emerging role in energy harvesting applications, particularly in enhancing the efficiency and stability of nanogenerators, highlights a critical area of growth. Overall, this review highlights the transformative potential of silane modification in advancing NM science and engineering, offering tailor-made solutions for diverse technological challenges.

## Data Availability

Not applicable.
